# Comprehensive analysis of the complete mitochondrial genomes of three *Coptis* species (*C. chinensis*, *C. deltoidea* and *C. omeiensis*): the important medicinal plants in China

**DOI:** 10.3389/fpls.2023.1166420

**Published:** 2023-05-29

**Authors:** Furong Zhong, Wenjia Ke, Yirou Li, Xiaoyan Chen, Tao Zhou, Binjie Xu, Luming Qi, Zhuyun Yan, Yuntong Ma

**Affiliations:** ^1^ State Key Laboratory of Characteristic Chinese Medicine Resources in Southwest China, Chengdu University of Traditional Chinese Medicine, Chengdu, China; ^2^ School of Pharmacy, Chengdu University of Traditional Chinese Medicine, Chengdu, China; ^3^ Innovative institute of Chinese Medicine and Pharmacy, Chengdu University of Traditional Chinese Medicine, Chengdu, Sichuan, China; ^4^ School of Health Preservation and Rehabilitation, Chengdu University of Traditional Chinese Medicine, Chengdu, Sichuan, China; ^5^ Key Laboratory of Traditional Chinese Medicine Regimen and Health, State Administration of Traditional Chinese Medicine, Chengdu, Sichuan, China

**Keywords:** *Coptis* species, mitochondrial genome, repeat sequence, genome size variation, thermal acclimation

## Abstract

*Coptis* plants (Ranunculaceae) contain high levels of isoquinoline alkaloids and have a long history of medicinal use. *Coptis* species are of great value in pharmaceutical industries and scientific research. Mitochondria are considered as one of the central units for receiving stress signals and arranging immediate responses. Comprehensive characterizations of plant mitogenomes are imperative for revealing the relationship between mitochondria, elucidating biological functions of mitochondria and understanding the environmental adaptation mechanisms of plants. Here, the mitochondrial genomes of *C. chinensis*, *C. deltoidea* and *C. omeiensis* were assembled through the Nanopore and Illumina sequencing platform for the first time. The genome organization, gene number, RNA editing sites, repeat sequences, gene migration from chloroplast to mitochondria were compared. The mitogenomes of *C. chinensis*, *C. deltoidea* and *C. omeiensis* have six, two, two circular-mapping molecules with the total length of 1,425,403 bp, 1,520,338 bp and 1,152,812 bp, respectively. The complete mitogenomes harbors 68-86 predicted functional genes including 39-51 PCGs, 26-35 tRNAs and 2-5 rRNAs. *C. deltoidea* mitogenome host the most abundant repeat sequences, while *C. chinensis* mitogenome has the largest number of transferred fragments from its chloroplasts. The large repeat sequences and foreign sequences in the mitochondrial genomes of *Coptis* species were related to substantial rearrangements, changes in relative position of genes and multiple copy genes. Further comparative analysis illustrated that the PCGs under selected pressure in mitochondrial genomes of the three *Coptis* species mainly belong to the mitochondrial complex I (NADH dehydrogenase). Heat stress adversely affected the mitochondrial complex I and V, antioxidant enzyme system, ROS accumulation and ATP production of the three *Coptis* species. The activation of antioxidant enzymes, increase of T-AOC and maintenance of low ROS accumulation in *C. chinensis* under heat stress were suggested as the factors for its thermal acclimation and normal growth at lower altitudes. This study provides comprehensive information on the *Coptis* mitogenomes and is of great importance to elucidate the mitochondrial functions, understand the different thermal acclimation mechanisms of *Coptis* plants, and breed heat-tolerant varieties.

## 1 Introduction

The constant increase in the accumulation of greenhouse gases has increasingly led to rise in average global temperature (Rivero et al., 2022). Temperature changes are closely related to plant growth and development. Medicinal plants exposed to high temperatures have impaired growth and medicine quality (Heydari et al., 2018; Kim et al., 2022). Global warming has also reduced the area of suitable habitats for medicinal plants that grow at higher altitudes (e.g. *Panax notoginseng*) (Zhan et al., 2022). Therefore, to maintain the sustainable use of medicinal resources in the face of climate change, it is important to understand the mechanisms of differential adaptation to high temperatures in medicinal plants and to obtain genetic resources for heat tolerance.

As pharmaceutically and economically important plants, *Coptis* genus (Ranunculaceae) mainly distributes in eastern Asia and North America (Xiang et al., 2018). Five *Coptis* species (*Coptis chinensis* Franch., *C. deltoidea* C. Y. Cheng et Hsiao, *C. teeta* Wall., *C. omeiensis* (Chen) C. Y. Cheng, and *C. quinquesecta* W. T. Wang) are distributed in southern and southwestern China. The dried rhizomes of *Coptis* plants named Coptidis Rhizoma with definitive pharmacological roles in anti-inflammatory, anti-bacteria, anti-diabetic and neuroprotection have a long history of medicinal use in China (Li et al., 2019; Ran et al., 2019; Wang et al., 2019). Coptidis Rhizoma is rich in berberine (Qi et al., 2018) which shows considerable potential as resource with various pharmacological activities for pharmaceutical and healthcare market (Song et al., 2020). *C. chinensis*, *C. deltoidea* and *C. teeta* are the sources of Coptidis Rhizoma stipulated in Chinese Pharmacopoeia, while the other *Coptis* species have been used as alternative folk herbs. The three Pharmacopeia-recorded *Coptis* species have been cultivated on different scales for several hundred years, while *C. omeiensis* has not been artificially cultivated (Mukherjee and Chakraborty, 2019). In China, the wild populations of these species are restricted distribution and almost endangered. The three closely related *Coptis* species exhibit significant differences in the altitudinal distribution ranges (http://www.iplant.cn/frps). The high temperatures in summer are one of the most important factors affecting the survival and herb production of *Coptis* plants and global warming will reduce the suitable growth areas of *Coptis* species (Li et al., 2020). *C. chinensis* with stronger environmental adaptability has a wider distribution range and become a widely cultivated species nowadays (He et al., 2014). Nevertheless, the molecular and physiological mechanisms of the differences in the thermal adaptation of *Coptis* species are poorly understood and the breeding of heat-tolerant cultivars has not been carried out. Limitations of the growing environment and the lack of improved varieties have limited the cultivation and industrial development of *Coptis* species.

In plants, mitochondria are essential organelles and responsible for plant energy metabolism. Mitochondria are considered as one of the central units for receiving stress signals and arranging immediate responses (Rasmusson and Møller, 2011). To counteract the environment stress, plants require high energetic demand, and mitochondria produce energy through oxidative phosphorylation, which determines the efficiency of ATP production and the level of reactive oxygen species (ROS) production (Luo et al., 2013). Mitochondrial oxidative phosphorylation is considered to be an important metabolic participant in plants under environmental stress, since the ATP synthesis capacity of the mitochondria and mitochondrial complex V activity greatly varies according to environmental stress, such as temperature and salt (Rurek, 2014; Zancani et al., 2020). In addition, mitochondria ROS generation is particularly important in signaling systems that integrate energy metabolism and stress responses in plants (Waszczak et al., 2018). The mitochondrial electron transport chain is responsible for the production of mitochondria ROS, which is thought to generate at the inner mitochondrial membrane and is associated particularly with Complex I. The rapid accumulation of ROS occurred in plants when they exposed to heat and drought (Medina et al., 2021), which can severely cause oxidative damage to DNA, proteins, and membrane lipids. A considerable amount of work has demonstrated that the ROS scavenging mechanisms play an important role in plant tolerance to high temperature stress.

Comprehensive analysis of mitochondrial genome is important to reveal the function of plant mitochondria (Cheng et al., 2021). Comparing to the counterparts of animals and fungi, land plant mitochondrial genomes exhibit some unique features, such as extreme diversity in size (Levings and Brown, 1989; Rodríguez-Moreno et al., 2011), complex genome structures (Ogihara et al., 2005; Guo et al., 2016), numerous repetitive sequences of various sizes and numbers (Cole et al., 2018; Dong et al., 2018), multiple RNA editing modifications (Kovar et al., 2018; Small et al., 2020), and frequent gene losses and foreign DNA migration during evolution (Cole et al., 2018; Dong et al., 2020). The sizes of plant mitogenome range from 66 kb (*Viscum scurruloideum*) (Skippington et al., 2015) to 11.3 Mb (*Silene conica*) (Sloan et al., 2012). Compared with nuclear and chloroplast genomes in plants, most angiosperm mitochondrial genomes have meager base substitution rates (Drouin et al., 2008). In contrast, the mitochondrial genomes of angiosperms evolve very rapidly in terms of structural rearrangements and gene transfer (Gualberto and Newton, 2017). The significant variation and different gene orders may occur within closely related species (Van de Paer et al., 2018), for example, within *Cucumis* (Cucurbitaceae) (Xia et al., 2022), *Monsonia* (Geraniaceae) (Cole et al., 2018), *Mangifera* (Anacardiaceae) (Niu et al., 2022). The high complexity of the plant mitochondrial genome makes it difficult to sequence and assemble. With the development of sequencing technology, the assembly and annotation of plant organelle genomes are promoted (Shearman et al., 2016). Approximately 437 plant mitochondrial genomes have been assembled and submitted to NCBI GenBank, that shed new light on the structure features and genetic composition of plant mitogenomes (Bi et al., 2022). In recent years, more researches have shown that comparative analysis of plant mitochondrial genomes can reflect the genetic information and evolutionary relationships between different species (Wang et al., 2021; Bi et al., 2022; Fan et al., 2022). Plant mitochondrial genome mainly encodes genes related to respiratory metabolism and oxidative phosphorylation, and these genes coordinate with the nucleus to ensure the function of plentiful proteins involved in the mitochondrial respiratory chain and the metabolic adaptations of plants (Osellame et al., 2012). Therefore, comprehensive characterizations of plant mitogenomes are imperative for revealing the relationship between mitochondria, elucidating biological functions of mitochondria and understanding the environmental adaptation mechanisms of plants.

In this study, the complete mitochondrial genomes of *C. chinensis*, *C. deltoidea* and *C. omeiensis* were assembled and annotated. The gene content, codon usage, RNA editing sites, repeat sequences, genome rearrangement, gene transfer among chloroplast and mitochondrial genomes of three *Coptis* species were characterized. Mitogenomic synteny analysis and comparative analysis of plant mitochondrial genomes are helpful to understand mitochondrial DNA differentiation and evolution in *Coptis* species. Furthermore, we carried out preliminary research on the heat stress-induced responses of *C. chinensis*, *C. deltoidea* and *C. omeiensis*. The expression level of several genes of mitochondrial complex I and V in were three *Coptis* species under 5 d heat stress were compared. The variations in accumulation of ROS, ATP content, activities of the antioxidant enzymes and activities of complex I and V were also evaluated. This study revealed the genetic information of three *Coptis* mitogenomes, and will provide important genetic resources for cultivation and utilization of these pharmaceutical species, contribute to better understanding of the thermal acclimation mechanisms of *Coptis* plants and provide a basis for the identification and cultivation of germplasm resources with a high degree of heat tolerance.

## 2 Materials and methods

### 2.1 Plant materials

**Figure 1:**
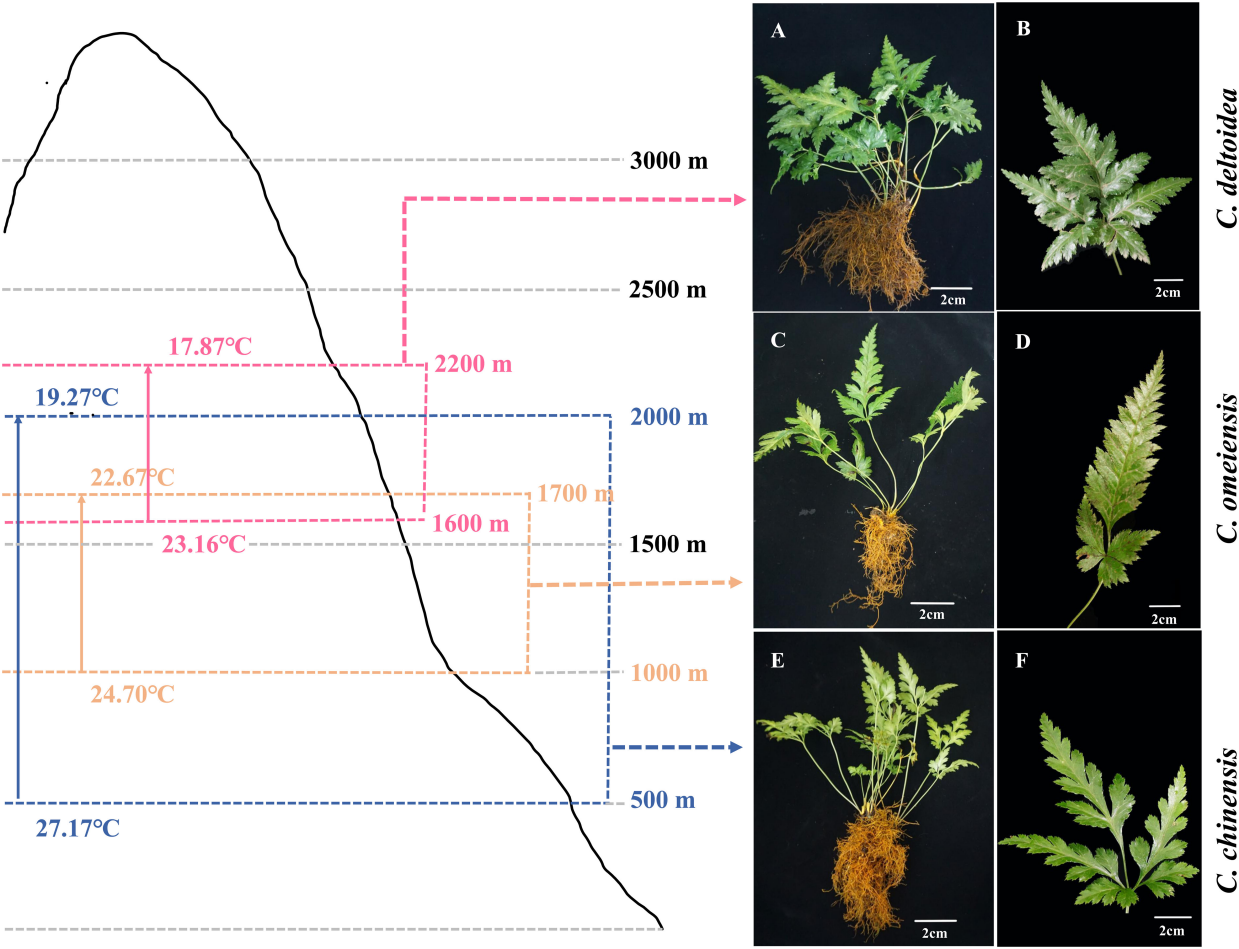
Distribution range and the plant morphology of *C. deltoidea* **(A, B)**, *C. omeiensis* **(C, D)** and *C. chinensis* **(E, F)**. Pink, orange and blue range represent *C. deltoidea*, *C. omeiensis*, and *C. chinensis*, respectively. The average summer temperatures (June-August) at different altitudes of the *Coptis* distribution areas were marked with different colors.

The *C. chinensis*, *C. deltoidea* and *C. omeiensis* plants (**Figure 1**) used in this study were grown in experiment field in Hongya, Sichuan, China (29°29′10.91″ N, 103°9′39.9″ E). Fresh leaves of the three *Coptis* plants were collected and immediately frozen in liquid nitrogen, then stored at -80°C.

### 2.2 DNA extraction, sequencing and assembly

High-quality genomic DNA was extracted from the leaves using a modified CTAB method (Porebski et al., 1997). DNA degradation and contamination were monitored on 1% agarose gels. After libraries construction using SQK-LSK109 (Nanopore Technologies), DNA sequencing was performed using Nanopore PromethION sequencing platform (Nanodrop Technologies, Wilmington, DE, US). NanoFilt and NanoPlot in Nanopack were used to filter and re-edit the raw data (De Coster et al., 2018). Meanwhile, the high-quality DNA from each sample was used to construct the libraries with average fragment length of 350 bp by the NexteraXT DNA Library Preparation Kit. Illumina Novaseq 6000 platform (Illumina, San Diego, CA, USA) was used to sequence the DNA samples and the raw sequencing data were edited using the NGS QC Tool Kit v2.3.3 (Patel and Jain, 2012). After trimming adapter sequences with Porechop (Wick et al., 2017a), Miniasm (Li, 2016) was applied to obtained a rough but computationally efficient assembly, which was then polished with Racon (Vaser et al., 2017). We selected contigs with homology to the *Hepatica maxima* (NCBI Sequence: MT568500.1) mitochondrial genome using Bandage (Wick et al., 2015). Retaining contigs with at least one ≥5 kb were aligned to the Hepatica maxima mitochondrion by BlastN. We then proceeded to align back the Nanopore reads to our draft assemblies of *C. chinensis*, *C. deltoidea* and *C. omeiensis* with minimap2 (Li, 2018). The aligned reads were *de novo* assembled with Unicycler (Wick et al., 2017b) and Flye (Kolmogorov et al., 2019). The final mitogenome sequences of *C. chinensis*, *C. deltoidea* and *C. omeiensis* were obtained by polishing with Pilon using Illumina Novaseq 6000 sequencing reads.

### 2.3 Mitogenomes annotation and sequence analysis

The mitogenomes of three *Coptis* species were annotated using the MITOFY webserver (http://dogma.ccbb.utexas.edu/mitofy/) (Alverson et al., 2010). The putative genes were manually checked and further adjusted according to relative species as the reference sequence. The tRNAs were annotated with the tRNA scan-SE software (http://lowelab.ucsc.edu/tRNAscan-SE/) with default settings (Schattner et al., 2005; Cheng et al., 2021). The relative synonymous codon usage (RSCU) values and amino acid composition of PCGs were calculated by MEGA X (Kumar et al., 2018). The circular maps of the *Coptis* mitochondrial genomes were visualized by using OrganellarGenomeDRAW (OGDRAW v1.3.1) (Greiner et al., 2019).

The possible RNA editing sites in the PCGs of *C. chinensis*, *C. deltoidea* and *C. omeiensis* mitogenomes were predicted using the PREP-Mt Web-based program (http://prep.unl.edu/) with the cut-off value set as 0.2 (Mower, 2005). Genome synteny and rearrangements among the three *Coptis* mitogenomes were analyzed following Guo et al. (Guo et al., 2016), implementing the progressiveMauve algorithm in Mauve ver. 2.4.0 software (Darling et al., 2004). KaKs_Calculator v.2.0 was used to calculate the synonymous (*Ks*) and nonsynonymous (*Ka*) substitution rates for all PCGs in three *Coptis* mitogenomes (Zhang et al., 2006).

### 2.4 Identification of repeat sequences and chloroplast gene insertion in *Coptis* mitogenomes

The position and type of SSR sequences were analyzed with the MISA-web (https://webblast.ipk-gatersleben.de/misa/) (Beier et al., 2017). The minimum thresholds of repeats were set to 8, 4, 4, 3, 3, and 3 for mono-, di-, tri-, tetra-, penta-, and hexanucleotides, respectively. The online program REPuter (https://bibiserv.cebitec.uni-bielefeld.de/reputer) (Kurtz et al., 2001) was used to detect forward (F), reverse (R), palindromic (P), and complement (C) repeats with a minimum repeat size of 30 bp and the repeat identity was > 90%.

To identify plastid derived mitochondrial sequences of three *Coptis* species, the mitogenomes were searched against the chloroplast genomes of *C. chinensis* (NC_036485), *C. deltoidea* (MT576696), *C. omeiensis* (NC_054330) using the BLASTN tool with the default settings. The circular maps of mitochondrial and chloroplast genomes and gene transfer segments of three *Coptis* species were visualized by using Circos v0.69 software.

### 2.5 Phylogenetic analysis

The 9 conserved mitochondrial PCG genes (*atp8*, *ccmB*, *ccmC*, *cob*, *cox1*, *cox3*, *nad3*, *nad4L* and *nad9*) from the three *Coptis* mitogenomes and 26 plant mitochondrial genomes were aligned using the MAFFT 7.037 software (Katoh and Standley, 2013). A maximum likelihood (ML) tree was constructed in MEGA 7.0 using the GTR+G+I nucleotide substitution model with 1000 bootstrap replicates. *Oryza sativa*, *Zea mays* and *Ginkgo biloba* were used as outgroups. To further reveal the phylogenetic relationships of the three *Coptis* species, a maximum likelihood (ML) tree of seven Ranunculaceae species (*Pulsatilla chinensis*, *P. dahurica*, *Anemone maxima*, *Aconitum kusnezoffii*, *C. chinensis*, *C. deltoidea* and *C. omeiensis*) and three outgroups (*Liriodendron tulipifera*, *Magnolia officinalis* and *M. biondii*) was constructed based on 21 conserved mitochondrial PCGs (*atp4*, *atp8*, *ccmB*, *ccmC*, *ccmFN*, *cob*, *cox1*, *cox3*, *mttB*, *nad1*, *nad2*, *nad3*, *nad4*, *nad4L nad5*, *nad7*, *nad9*, *rpl5*, *rpl16*, *rps13*, *sdh4*) using MEGA 7.0. The mitochondrial genomes used to perform phylogenetic analysis were listed in **Supplementary Table S1**.

### 2.6 High-temperature treatment and sampling

The *C. chinensis*, *C. deltoidea* and *C. omeiensis* plants were grown in pots (10*15 cm) containing paddy soil. The plants were divided into two groups and placed in growth chamber set at 20°C (control) and 30°C (heat treatment group) for 5 days, with 6 individuals in each treatment group. The relative humidity of the growth chamber was set at 85% and the light intensity was set at 100 μmol m^-2^ s^-1^ with an 8 h/16 h light/dark cycle. To determine physiological and biochemical indices and RNA extraction, fully expanded leaves with three biological replicates from three *Coptis* species were collected and frozen in liquid nitrogen immediately and then stored at -80°C.

### 2.7 Real-time quantitative PCR analysis

Total RNA was isolated from 50 mg of fresh leaves from control and heat treatment group by using HP plant RNA kit (Omega HP-Plant, USA) according to the manufacturer’s instructions. The cDNAs were synthesized with total RNA (0.5 μg) from different treatments following the instruction of Master Premix for first-strand cDNA synthesis (FOREGENE, Chengdu, China). RT-qPCR were performed using 2 μL of a tenfold dilution of the cDNA in 20 μL solution system composed of 2× Real PCR Easy™ Mix-SYBR (FOREGENE, Chengdu, China). The *18S* ribosomal RNA was used as an internal control gene for normalization (Ref) and the primers of each genes (*nad1*, *nad2*, *nad5* and *nad6*) are listed in **Supplementary Table S2**. The condition of PCR amplification reaction was conducted as follow: 95°C for 3 min, followed by 40 cycles of 95°C for 10 s and 61°C for 30 s. The relative expression levels of target genes were calculated using the 2^−ΔΔCt^ comparative threshold cycle (Ct) method.

### 2.8 Determination of superoxide anions, hydrogen peroxide, ATP content, activities of antioxidant enzymes, total antioxidant capacity and activities of mitochondrial complex I and V

The concentrations of superoxide anion and H_2_O_2_ were measured using detection assay kits (Solarbio, Beijing, China) following the manufacturer’s instructions. The ATP content was measured using phosphomolybdic acid colorimetry according to the ATP assay kit (G0815W96) purchased from Suzhou Grace Biotechnology Co. (Suzhou, China). For the enzyme assays, superoxide dismutase (SOD) activity was measured using a SOD detection kit (Solarbio, Beijing, China), and catalase (CAT) activity was measured following the instruction of CAT detection kit (Solarbio, Beijing, China), and peroxidase (POD) activity was detected with a POD detection kit (Solarbio, Beijing, China). T-AOC, mitochondrial complex I and V activities were measured by T-AOC assay kit, complex I assay kit and complex V (Solarbio, Beijing, China).

### 2.9 Statistical analysis

Data from all treatments were subjected to analysis of variance (ANOVA) by the SPSS statistical software package version 25 (SPSS Inc., USA). Significant differences between treatment means were identified by Student’s *t*-test and Duncan’s multiple range test at the *p* < 0.05. Origin (Origin 2019, USA) was used for figure construction.

## 3 Results

### 3.1 A panel of three complete *Coptis* mitogenomes and basic characteristics

**Figure 2:**
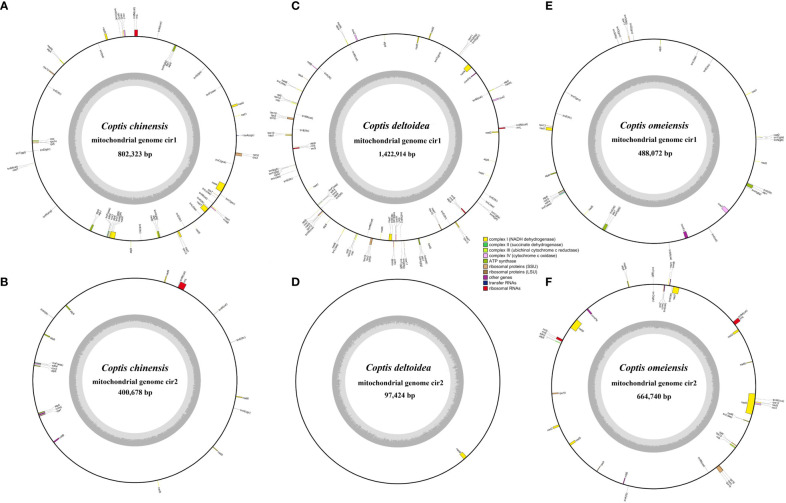
Circular gene map of three *Coptis* mitogenomes. **(A, B)** The two large molecules of *C. chinensis* mitogenome. **(C, D)** The circular map of two molecules of *C. deltoidea* mitogenome. **(E, F)** The circular map of two molecules of *C. omeiensis* mitogenome. Genes shown on the outside and inside of the circle are transcribed clockwise and counterclockwise, respectively. The dark gray region in the inner circle represents GC content.

Based on analysis of DNA sequences generated from high-throughput sequencing, we performed mitochondrial genome assemblies of three *Coptis* species (**Supplementary Tables S3**, **S4** and **Supplementary Figure S1**) and investigated the divergence of genome-wide sequence. The mitochondrial genome sizes of *C. chinensis*, *C. deltoidea* and *C. omeiensis* are 1,425,403 bp, 1,520,338 bp and 1,152,812 bp, respectively (**Table 1**), which are similar to the reported mitochondria of *Anemone maxima* (1,122,546 bp) (Park and Park, 2020) and are longer than the mitochondrial sequences of *Aconitum kusnezoffii* (440,720 bp) (Li et al., 2021), which also belong to in the Ranunculaceae species with reported mitochondrial sequences. The mitogenome of *C. chinensis* comprises six circular molecules with the size from 26,641 to 802,323 bp (**Figures 2A, B**; **Supplementary Figure S2**), whereas the *C. deltoidea* mitogenome was assembled into two circular molecules with sizes of 1,422,914 and 97,424 bp (**Figures 2C, D**), and the assembled mitochondrial genome of *C. omeiensis* is two complete circular scaffolds with the length of 488,072 and 664,740 bp (**Figures 2E, F**; **Supplementary Table S5**). The fragments migrated from the chloroplast genome accounted for a variable proportion (4.28%, 2.34% and 2.07%) of the three *Coptis* mitochondrial genomes, with the highest proportion in *C. chinensis* mitogenome (**Supplementary Figure S3**).

**Table 1:** Genomic features of *Coptis* mitochondrial genomes.

Genome feature	*C. chinensis*	*C. deltoidea*	*C. omeiensis*
Genome size (bp)	1,425,403	1,520,338	1,152,812
GC (%)	45.7	45.8	45.8
Number of genes	84	86	68
Number of tRNAs	35	30	26
Number of rRNAs	2	5	3
Number of PCGs	47	51	39
Cis-spliced introns	16	16	15
Number of large repeats (> 1 kb)	22	31	4
Total length of PCGs (bp)	37,224 (2.61%)	36,273 (2.38%)	31,878 (2.77%)
Total length of tRNAs (bp)	2,571 (0.18%)	2,229 (0.15%)	1,925 (0.17%)
Total length of rRNAs (bp)	7,522 (0.53%)	6,877 (0.45%)	6,015 (0.52%)
Total length of cis-spliced introns (bp)	31,053 (2.18%)	29,749 (1.96%)	26,311 (2.28%)
Mitochondrial DNA of chloroplast origin (bp)	61,038 (4.28%)	35,596 (2.34%)	23,847 (2.07%)
Gross length of long repeats (bp)	293,491 (20.59%)	692,208 (45.53%)	78,461 (6.81%)

Overall guanine-cytosine (GC) content of the three *Coptis* species are 45.7%, 45.8% and 45.8% respectively (**Supplementary Table S5**), indicating mitochondrial DNA similarity between these species and other angiosperms. The three complete *Coptis* mitogenomes encode 68-86 predicted functional genes, including 39-51 protein-coding genes (PCGs), 26-35 transfer RNA (tRNA) genes specifying 16-20 amino acids, and 2-5 ribosomal RNA (rRNA) genes and share nearly intron content accounting for 2.18%, 1.96%, and 2.28% of the *Coptis* mitochondrial genomes (**Table 1**). Most PCGs have no introns, however, nine genes (*nad1*, *nad2*, *nad4*, *nad5*, *nad7*, *cox2*, *ccmFC*, *rps3*, *rps10*) are found to contain one or more introns in three *Coptis* mitochondrial genomes (**Table 2**). The distribution of amino acid residues across the mitochondrial proteins share very highly similarity in Ranunculaceae plants (**Supplementary Figure S3**). Moreover, a total of 637, 605 and 653 RNA editing sites is predicted in PCGs of *C. chinensis*, *C. deltoidea* and *C. omeiensis* respectively (**Supplementary Figure S4** and **Supplementary Table S6**). Among the functionally different genes, respiratory complex I (NADH dehydrogenase) genes, cytochrome C biogenesis genes and transport membrance protein (mttB) exhibit the largest average numbers of editing sites (**Supplementary Figure S4**).

**Table 2:** Gene profile and organization of the *C. chinensis*, *C. deltoidea* and *C. omeiensis* mitogenomes.

Group of genes	*C. chinensis*	*C. deltoidea*	*C. omeiensis*
Complex I (NADH dehydrogenase)	*nad1* ^*^, *nad*2^***^, *nad3*, *nad4* ^***^, *nad4L*, *nad5* ^*^, *nad6*(3), *nad7* ^****^, *nad9*	*nad1* ^*^(2), *nad2* ^*^, *nad3*, *nad4* ^***^, *nad4L*(2), *nad5* ^*^, *nad6*(2), *nad7* ^****^, *nad9*	*nad1* ^*^, *nad2* ^**^, *nad3*, *nad4* ^***^, *nad4L*, *nad5* ^*^(2), *nad6*, *nad7* ^****^, *nad9*
Complex II (succinate dehydrogenase)	*sdh4*(2)	*sdh4*	*sdh3*, *sdh4*
Complex III (ubichinol cytochrome c reductase)	*cob*	*cob*(2)	*cob*
Complex IV (cytochrome c oxidase)	*cox1*, *cox3*(2)	*cox1*, *cox2* ^*^(2), *cox3*	*cox1*, *cox2* ^*^, *cox3*
Complex V (ATP synthase)	*atp1*(3), *atp4*, *atp6*, *atp8*(2), *atp9*(5)	*atp1*, *atp4* (2), *atp8*, *atp9* (6)	*atp1*(2), *atp4*, *atp6*, *atp8*, *atp9*(2)
Cytochrome c biogenesis	*ccmB*, *ccmC*, *ccmFc* ^*^, *ccmFN*	*ccmB*, *ccmC*, *ccmFN*	*ccmB*, *ccmC*, *ccmFc* ^*^, *ccmFN*
Small subunit ribosomal proteins (SSU)	*rps3* ^*^(2), *rps7*(2), *rps10* ^*^, *rps11*, *rps12*, *rps13*, *rps14*, *rps19*	*rps3* ^*^(2), *rps4*, *rps7*, *rps10* ^*^, *rps11*(2), *rps12*, *rps13*(2), *rps14*(2), *rps19*(2)	*rps3* ^*^, *rps4*, *rps7*, *rps10* ^*^, *rps11*, *rps12*, *rps13*, *rps14*, *rps19*
Large subunit ribosomal proteins (LSU)	*rpl5*, *rpl16*(2)	*rpl5*(2), *rpl16*(2)	*rpl5*, *rpl16*
Transfer RNAs	*trnA-CGC*(2), *trnC-GCA*(2), *trnD-GTC*, *trnE-TTC*(4), *trnF-AAA*(3), *trnfM-CAT*, *trnG-GCC*, *trnH-GTG*(2), *trnI-CAT*, *trnI-TAT*, *trnK-TTT*(2), *trnL-CAA*, *trnL-TAA*, *trnM-CAT*(5), *trnN-GTT*, *trnQ-TTG*, *trnR-ACG*, *trnS-TGA*, *trnT-GGT*, *trnV-GAC*, *trnW-CCA*, *trnY-GTA*	*TrnC-GCA*, *trnD-GTC*, *trnE-TTC*(4), *trnF-AAA*, *trnfM-CAT*(2), *trnG-GCC*, *trnH-GTG*, *trnI-TAT*, *trnK-TTT*(3), *trnL-TAA*(4), *trnM-CAT*(3), *trnN-GTT*(2), *trnQ-TTG*(2), *trnT-GGT*, *trnW-CCA*, *trnY-GTA*(2)	*TrnC-GCA*, *trnD-GTC*, *trnE-TTC*(2), *trnF-AAA*, *trnfM-CAT*(2), *trnG-GCC*(2), *trnH-GTG*(2), *trnI-TAT*(2), *trnK-TTT*(4), *trnL-TAA*(2), *trnM-CAT*, *trnN-GTT*, *trnQ-TTG*(2), *trnT-GGT*, *trnW-CCA*, *trnY-GTA*
Ribosomal RNAs	*rrnL*(2)	*rr5*(2), *rrL*, *rrS*(2)	*rrn5*, *rrnL*, *rrnS*
Transport membrane protein	*mttB*	*mttB*	*mttB*

^*^for introns, and the number of ^*^represent the number of introns. For example, ^***^represents the number of three introns. Gene(2): The numbers after the gene names indicate the number of copies of multi-copy genes. For example, nad6(2) represents that two copies of nad6 are found in the mitogenome.

### 3.2 Repeats and structural variation among the three *Coptis* mitochondrial genomes

**Figure 3:**
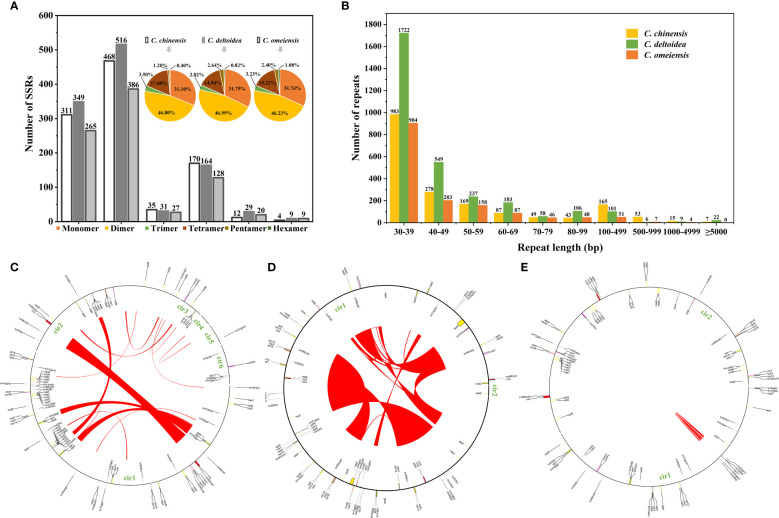
Detected repeats in the mitochondrial genomes of *C. chinensis*, *C. deltoidea* and *C. omeiensis*. **(A)** Type and proportion of detected SSRs. **(B)** Length distribution of long repeats in the *Coptis* mitochondrial genomes. Histograms display the number of repeats with given lengths. **(C–E)** The locations of the large repeats (>1,000 bp) in the mitochondrial genomes of three Coptis species.

Simple sequence repeats (SSRs) are sequence-repeating units with 1-6 nucleotides. 1,000, 1,098, and 835 SSRs were identified in the mitochondrial genomes of *C. chinensis*, *C. deltoidea* and *C. omeiensis*, and **Figure 3A** displays the proportion of each kind. Among the three *Coptis* species, dimer repeats comprised 46.23- 46.99% of the total, followed by monomer repeats at 31.10-31.79%. Further analysis of the repeat unit of SSRs indicated that dinucleotide repeats (AG/CT) were more prevalent than the other repeat types. Based on the REPuter, we identified 1,849, 2,993 and 1,508 long repeats (> 30 bp) belonging to forward repeats in the mitogenomes of *C. chinensis*, *C. deltoidea* and *C. omeiensis*. The total length of the long repeats was 293,491 bp, 692,208 bp and 78,461 bp, accounting for 20.59%, 45.53% and 6.81% of the entire mitogenomes, respectively. Most repeats were 30–49 bp long, whereas 22, 31 and 4 repeats were longer than 1,000 bp in the three *Coptis* species, respectively (**Figure 3B**). The largest repeats were 39,327 bp, 150,222 bp and 2,270 bp in the mitogenomes of *C. chinensis*, *C. deltoidea* and *C. omeiensis* (**Figures 3C–E**).

**Figure 4:**
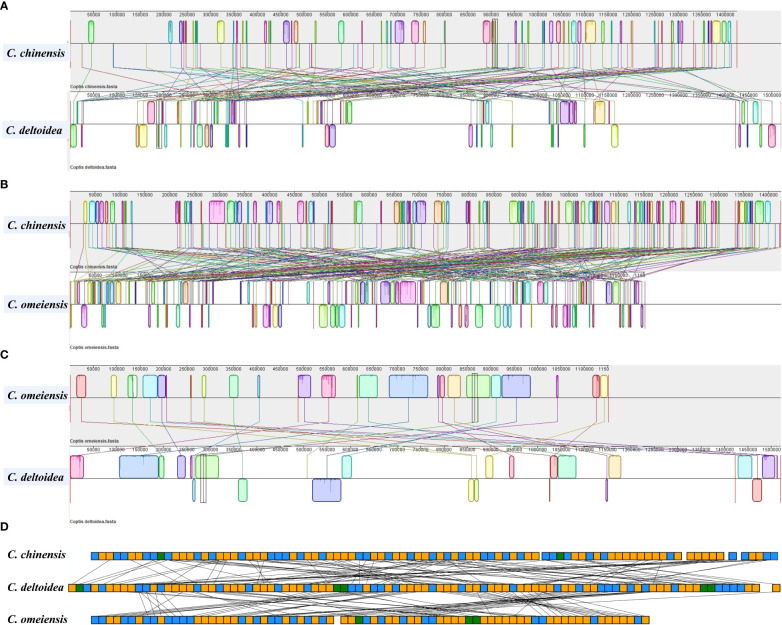
Colinearity and rearrangement analysis of *C. deltoidea*, *C. omeiensis*, and *C. chinensis*. **(A–C)** Mauve comparison of the three *Coptis* mitogenomes. **(D)** Relative order of PCGs in the three *Coptis* species. The orange, blue and green blocks represent CDS, tRNA and rRNA, respectively.

To further investigate structural variations in *Coptis* mitogenomes, the mitochondrial genome rearrangement and collinearity of *C. chinensis*, *C. deltoidea* and *C. omeiensis* were compared using the Mauve programs. Synteny analysis showed that there were many homologous regions among the three *Coptis* species (**Supplementary Table S7**). A total of 246 synteny blocks were found between *C. chinensis* and *C. omeiensis* mitogenomes. And relatively long synteny blocks were found between *C. deltoidea* and *C. omeiensis* mitogenomes with the longest block being 62,375 bp in length. The relative positions of these homologous regions were different, indicating abundant rearrangements and inversions within the three mitogenomes which were also related to the differences in relative order of genes in the three *Coptis* species (**Figure 4**). Different amounts and sizes of the locally collinear blocks and the expansion or contraction of regions between homologous sequences promoted the size dynamics of the *Coptis* mitogenomes.

### 3.3 Phylogenetic analysis, gene loss and multi-copy gene of three *Coptis* mitogenomes

**Figure 5:**
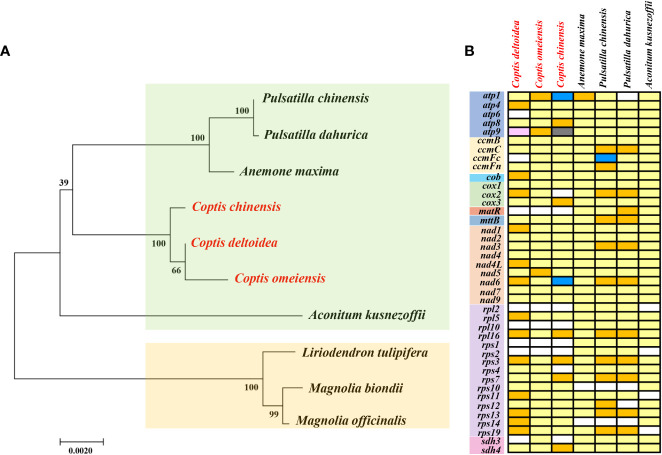
The phylogenetic relationships **(A)** and PCGs distribution of three *Coptis* species and other 4 Ranunculaceae species **(B)**. The Maximum Likelihood tree was constructed based on the sequences of 23 conserved protein-coding genes. Outgroup: *Liriodendron tulipifera*, *Magnolia officinalis* and *M. biondii*. About PCGs distribution, white boxes indicate that the gene is not present in the mt genomes. Light yellow, golden, blue, black and pink boxes indicate that one, two, three, five and six copies exist in the particular mitochondrial genomes, respectively.

Mitochondrial genomes provide important genetic information for phylogenetic studies of different species (Xia et al., 2022). The ML tree inferred from 9 conserved mitochondrial PCG genes of 29 species from 19 families revealed that six Ranunculales species (*Liriodendron tulipifera*, *Aconitum kusnezoffii*, *Anemone maxima*, *C. chinensis*, *C. deltoidea*, and *C. omeiensis*) clustered closely together into one clade (**Supplementary Figure S6**). To further reveal the phylogenetic relationships of the three *Coptis* species, a phylogenetic tree was constructed with three Magnoliaceae plants as outgroups (**Figure 5A**). It also strongly supports the closely related phylogenetic relationship of three *Coptis* spcies.

Mitochondrial gene content is highly variable across extant angiosperm and the loss of PCGs occurs frequently, even in closely related species (Adams and Palmer, 2003). In comparison with the 41 protein-coding genes inferred to be present in the ancestral mitogenome of angiosperms (Guo et al., 2016), the *matR*, *rpl2*, *rpl10*, *rps1* and *rps2* were lost in *C. chinensis*, *C. deltoidea* and *C. omeiensis* species (**Figure 5B**). In most land plants, the ribosomal proteins, cytochrome c biogenesis and succinate dehydrogenase genes were highly variable. The high diversity in the gene content among different angiosperm mitochondrial genomes was attributed to the variety of ribosomal protein genes (Liao et al., 2018). In addition, *sdh3* was lost in *C. chinensis* and *C. deltoidea*, and the *cox2* and *rps4* were lost in *C. chinensis* mitochondrial genome, whereas the *atp6* and *ccmFc* were lost in *C. deltoidea*. Overall, *C. omeiensis* has the relatively complete PCGs among the three *Coptis* species (**Figure 5B**).

Multi-copy genes are pervasive in plant mitochondrial genome (Fang et al., 2021). In *C. chinensis* mitogenome, *atp8*, *cox3* and *sdh4* were located in the large repeat R7 (11,718 bp), which creates duplicate copies of these genes. And three copies of *atp1* and two copies of *atp9* are associated with large repeat sequences R2 (16,570 bp) and R5 (15,647 bp). In addition, *rpl16, rps3, rps7* were also duplicated in *C. chinensis* mitogenome (**Figure 4C**). In *C. deltoidea* mitogenome, the genes including *atp4*, *cob*, *cox2*, *nad1*, *nad4L*, *nad6*, *rpl5*, *rpl16*, *rps3*, *rps11*, *rps13*, *rps14*, *rps19* are duplicated by the large repeats of R1, R2, R5 and R8 (**Figure 4D**). Whereas, only *atp1*, *atp9* and *nad5* were duplicated in *C. omeiensis* mitogenome (**Figure 5B**).

### 3.4 PCGs under selected pressure in *Coptis* mitochondrial genomes mainly related to the mitochondrial respiratory chain

The nonsynonymous-to-synonymous substitution ratio (*Ka*/*Ks*) is used to reflect the selective pressure and the evolutionary dynamics of PCGs. In this study, the *Ka*/*Ks* ratio was determined by 36 PCGs in *C. chinensis*, *C. deltoidea* and *C. omeiensis* (**Table 3**). The PCGs shared between *C. deltoidea* and *C. omeiensis* were close homologs, as the *Ka* and *Ks* ratio of 27 PCGs was 0. The genes with sequence differences included *atp9*, *nad1*, *nad2*, *nad5* and *rps3*. Among them, the *Ka*/*Ks* ratios of *nad*1, *nad*2 and *rps*3 were greater than 1, which indicated these genes had been under positive selection during evolution. Differences in some PCGs were found when comparing *C. chinensis* mitogenome to that of *C. deltoidea* and *C. omeiensis*. The *Ka*/*Ks* ratios of *atp1*, *atp6*, *atp9*, *nad5*, *nad6*, *rpl5*, *rps3*, *rps7*, *rps11* and *rps19* were less than 1, indicating that these PCGs were subject to purified selection during evolution, which may play vital roles in stabilizing the normal function of mitochondria (Betrán et al., 2006). These results indicated that the PCGs under selected pressure in *Coptis* mitochondrial genomes mainly belong to NADH dehydrogenase, ATP synthase and ribosomal proteins, which are closely related to the mitochondrial respiratory chain. In addition, the *Ka*/*Ks* ratios of *nad1* and *nad2* were greater than 1, indicating that *nad1* and *nad2* gene in *C. chinensis* mitogenome were under positive selection.

**Table 3:** Ka/Ks ratios of PCGs in C. chinensis, C. deltoidea and C. omeiensis.

Gene	*Ka/Ks* (*C. deltoidea vs C. omeiensis*)	*Ka/Ks* (*C. deltoidea vs C. chinensis*)	*Ka/Ks* (*C. chinensis vs C. omeiensis*)
*atp1*	NaN	0.7089	0.7089
*atp9*	0.0633	0.3634	0.3703
*nad1*	1.1793	0.0000	1.0506
*nad2*	1.5681	1.3961	1.0443
*nad5*	0.5787	0.2194	0.3846
*nad6*	NaN	0.8762	0.8762
*nad7*	NaN	*	*
*rpl5*	NaN	0.4374	0.4374
*rps3*	2.9681	0.5424	0.4996
*rps7*	NaN	0.1296	0.1296
*rps10*	NaN	*	*
*rps11*	NaN	0.1445	0.1445
*rps19*	NaN	0.4182	0.4182
*sdh4*	NaN	*	*

When Ks = 0, the value cannot be calculated, it was represented by *. When Ka = 0 and Ks = 0, it was represented by NaN.

### 3.5 The expressions of gene members of NADH dehydrogenase which were under selection pressure and the activities of complex I and complex V and under heat stress

**Figure 6:**
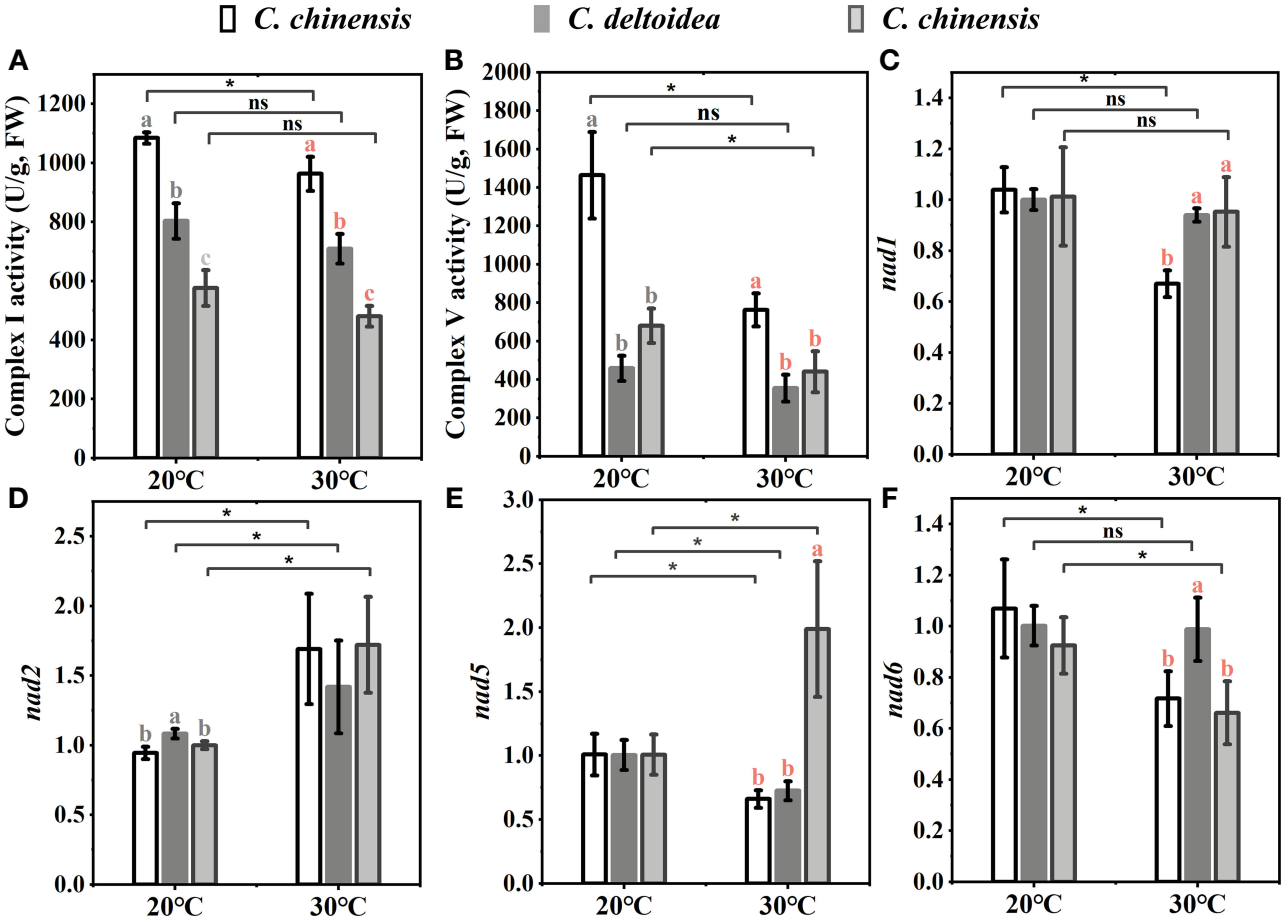
The activities of complex I **(A)** and complex V **(B)**, and the expressions of some PCGs involved in oxidative phosphorylation **(C–F)** of three *Coptis* species under 20°C and 30°C. Different letters with different color (gray and red) indicate a significant difference at *P* < 0.05 between three *Coptis* species under 20°C and 30°C, respectively. The asterisks indicate a significant difference between plants grown at 20°C and 30°C as determined by Student’s *t*-test (*p < 0.05, **p < 0.01) and ns means no difference between the treatment. Each column represents the mean ± standard deviation of three replicates.

In China, *C. deltoidea* grows mainly in the montane understory at altitudes between 1600 and 2200 m, while *C. omeiensis* is mainly distributed at altitudes between 1000 and 1700 m (**Figure 1**). Differently, the *C. chinensis* grows widely at altitudes from 500 to 2000 m. The three *Coptis* species exhibit significant differences in the altitudinal distribution ranges, indicating differences in thermo-tolerance. Mitochondria are dynamically involved in the stress response and key agents in how plants respond to oxidative stress (Jacoby et al., 2012). To preliminary characterize the biological functions of mitochondria in the environmental adaptation of *C. chinensis*, *C. deltoidea* and *C. omeiensis*, we compared the expressions of members of NADH dehydrogenase (*nad1*, *nad2*, *nad5* and *nad6*) which were under selection pressure and activities of complex I and complex V of three *Coptis* species grown at 20°C and 30°C for 5 d. The high-temperature stress increased expression levels of *nad2* and decreased expression levels of *nad6* in three *Coptis* species. Compared with 20°C, *nad1* expression in *C. chinensis* was significantly reduced under 30°C treatment, while the expression levels of *nad1* in *C. deltoidea* and *C. omeiensis* were not significantly changed. Moreover, the activity of complex I (1,084.65 ± 19.40 U/g) and complex V (1,463.89 ± 225.56 U/g) of *C. chinensis* were maintained at a higher level compared to that of *C. deltoidea* and *C. omeiensis* at 20°C (**Figures 6A, B**). Meanwhile, the activity levels of complex I and complex V were found to decrease under heat stress (i.e. 30°C) in the three *Coptis* species, with the activities of complex I and complex V of *C. chinensis* remaining significantly higher than those of *C. deltoidea* and *C. omeiensis* (**Figure 6**).

### 3.6 Concentration of superoxide anion, H_2_O_2_ and ATP, and antioxidant enzyme activity under heat stress

**Figure 7:**
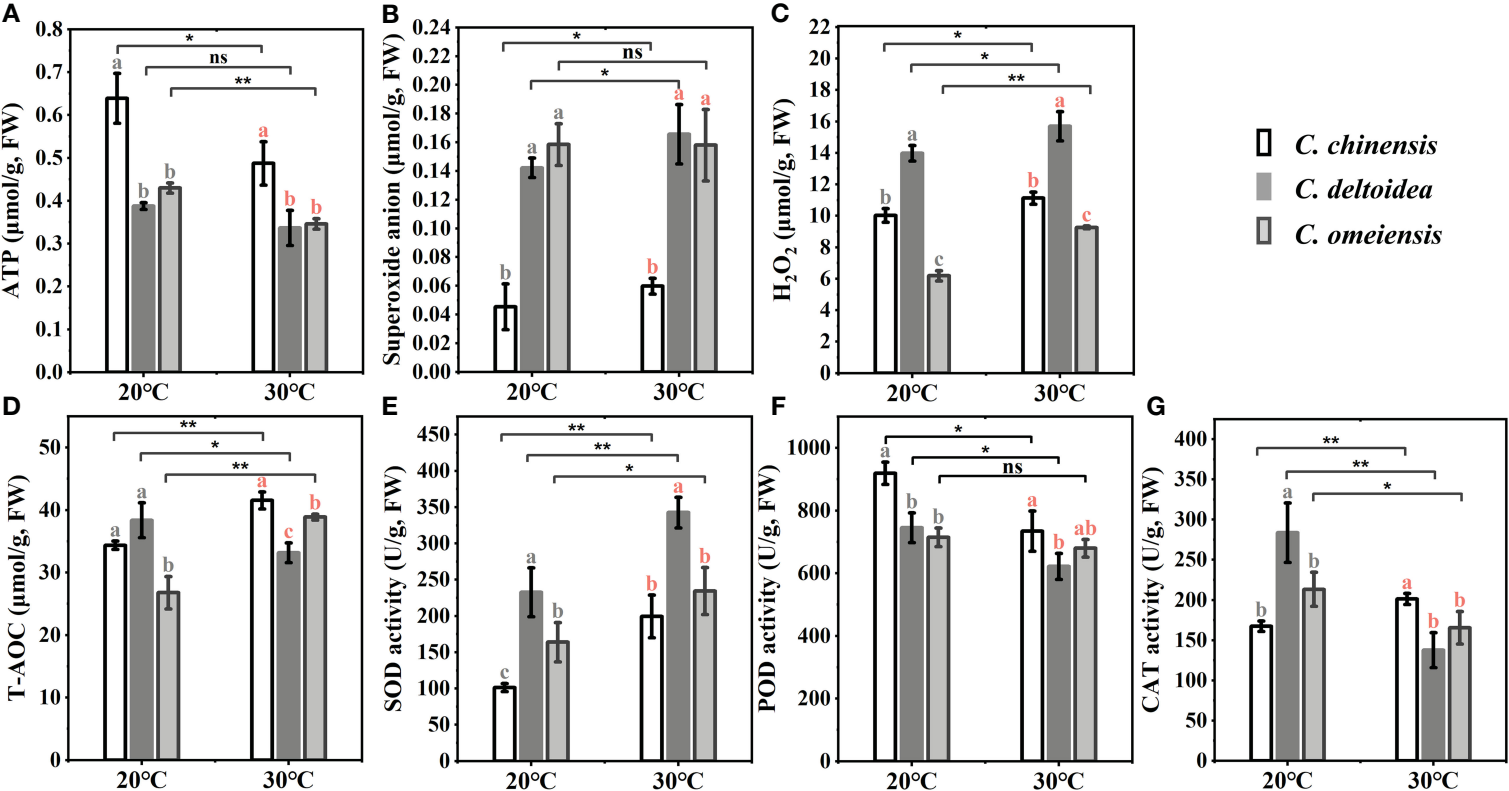
The concentration of superoxide anion, H_2_O_2_, ATP and T-AOC **(A–D)** and the activities of SOD, POD and CAT **(E–G)** of three *Coptis* species under 20°C and 30°C. Different letters with different color (gray and red) indicate a significant difference at *P* < 0.05 between three *Coptis* species under 20°C and 30°C, respectively. The asterisks indicate a significant difference between plants grown at 20°C and 30°C as determined by Student’s *t*-test (*p < 0.05, **p < 0.01) and ns means no difference between the treatment. Each column represents the mean ± standard deviation of three replicates.

The mitochondrial DNA-encoded electron transport chain (ETC) complex and ATP synthase are closely linked to the production of ROS and ATP, which play important roles in plant resistance to stress. we found that the ATP content of *C. chinensis* was significantly higher than that of *C. deltoidea* and *C. omeiensis* at 20 °C, while the ATP content of *C. omeiensis* was slightly higher than that of *C. deltoidea*, but the significant difference was not found. The decreased level of ATP in *C. chinensis*, *C. deltoidea* and *C. omeiensis* under heat stress was noticed, and the ATP concentration of *C. chinensis* remained significantly higher than that of *C. deltoidea* and *C. omeiensis* (**Figure 7**). Meanwhile, the content of superoxide anion and H_2_O_2_ at low levels in *C. chinensis* at 20°C. High temperature stress caused a significant increase in ROS levels of the three *Coptis* species. The ROS levels of *C. deltoidea* were significantly higher than those of *C. chinensis* and *C. omeiensis*. The SOD and CAT activities and T-AOC (total antioxidant activity) of *C. chinensis* at 20 °C were lower than those of *C. deltoidea* and *C. omeiensis*. In three *Coptis* species, SOD activity was stimulated, while POD activity was depressed by heat stress (**Figure 7**). A significant increase was detected in CAT activity in *C. chinensis* and a decrease was noted in those in *C. deltoidea* and *C. omeiensis* as compared to the plants under normal temperature (**Figure 7**). Moreover, the concentration of T-AOC increased significantly in *C. chinensis* and *C. omeiensis*, while the T-AOC decreased significantly in *C. deltoidea*, and the T-AOC of *C. chinensis* under heat stress was more stimulated than that of *C. deltoidea* and *C. omeiensis* (**Figure 7**).

## 4 Discussion

Mitochondria are the respiration sites of plants, which generate the ATP needed for cell maintenance and growth (Millar et al., 2011). Mitochondrial genome analysis is of great importance for understanding the biological functions of mitochondria, molecular evolution, genome structure and phylogenetic relationships of different plant species, especially closely related species (Niu et al., 2022; Li et al., 2023). As pharmaceutically and economically important plants, *C. chinensis*, *C. deltoidea* and *C. omeiensis* have different distribution ranges and requirements for environmental conditions. Under the extreme high temperature weather in summer, most leaves of *C. deltoidea* at the altitude of 1800 m were withered, while *C. chinensis* grew well (**Supplementary Figure 7**). Therefore, to preliminary reveal the differential mechanism of thermal acclimation, this study is the first to comprehensively analyze the complete mitochondrial genomes of three *Coptis* species.

### 4.1 Mitochondrial genome comparison and variation of *C. chinensis*, *C. deltoidea* and *C. omeiensis*

Mitogenomes of *C. chinensis*, *C. deltoidea* and *C. omeiensis* are conserved in GC content (45.7%, 45.8% and 45.8%) but differ substantially in genome size (1,425,403 bp, 1,520,338 bp and 1,152,812 bp), composition, and structure. The size variations in plant mitogenomes are primarily attributed to the proliferation of repeats, the migration of foreign sequences, and gain or loss of large intragenetic segments (Gualberto and Newton, 2017; Wu et al., 2022). Plant mitochondrial genomes have abundant non-tandem repeats (Wynn and Christensen, 2019). Previous study of several mitogenomes of Fabaceae species found that their size varied from 271,618 bp to 729,504 bp with large proportion of repeats (2.9–60.6%) accounting for most of the size variations (Choi et al., 2019). Positive correlations between mitogenome size and repeat content were identified in Rosaceae species (Sun et al., 2022). In our study, the proportion of long repeat sequences (>30 bp) in the *C. deltoidea* mitogenome (45.53%) was higher than that of *C. chinensis* (20.59%) and *C. omeiensis* (6.81%). These repeats may have contributed to the increase in the mitogenome size of *C. deltoidea* (bp). However, the mitogenome size of *C. chinensis* was close to that of *C. deltoidea*, which means that the mitogenome size is by no means only determined by repeats. The foreign sequences originating from chloroplast, nuclear, and mitochondrion of other species also contribute to the variable mitogenome size (Goremykin et al., 2012; Rice et al., 2013). Furthermore, large repetitive sequences and mitogenome expansion might led to multi-copy genes in plant mitogenomes (Miao et al., 2022). In *C. deltoidea* mitogenome, 16 large repeat sequences (>10,000 bp) were found. Among them, R1, R2, R5 and R8 were related to the duplicate copies of genes belonging to NADH dehydrogenase (*nad1*, *nad4L* and *nad6*), ubichinol cytochrome c reductase (*cob*), complex IV (*cox1* and *cox2*), cytochrome c oxidase (*cox2*), ATP synthase (*atp4*) and small subunit ribosomal proteins (*rps3, rps11, rps14, rps19*). In *C. chinensis* mitogenome, three copies of *atp1* and two copies of *atp8*, *atp9*, *cox3* and *sdh4* were also attributed to large repeats.

Although the phylogenetic analysis and synteny analysis indicated the closely related relationship and many homologous regions among the three *Coptis* species, large-scale gene rearrangements were detected in the mitochondrial genome of *C. chinensis*, *C. deltoidea* and *C. omeiensis*. In angiosperms, the accumulation of repeat sequences and repeat-mediated inversion are the predominant mechanism of rearrangement in mitogenome (Cole et al., 2018). The abundant rearrangements and significant variation in gene orders among these closely related *Coptis* species may be attributed to repeat sequences that were widely present in the mitogenomes.

*Ka/Ks* analysis of mitochondrial genome from the three *Coptis* species showed that the PCGs shared between *C. deltoidea* and *C. omeiensis* were close homologs. In addition, most of the PCGs in the three *Coptis* species were subject to purified selection as Ka/Ks ratios were less than 1.0, with *nad5* and *nad6* having relatively high *Ka/Ks* ratios. However, we also found genes in which the *Ka/Ks* >1, such as *nad1, nad2* and *rps3*. The results indicated that several members of NADH dehydrogenase (complex I) had been under pressure selection during evolution. Mitochondrial complex I is the first and largest respiratory complex required in the process of the process of oxidative phosphorylation to generate ATP, which is involved in the mitochondrial electron transport chain and is one of the major production sites of ROS (superoxide anion) (Huang et al., 2016; Subrahmanian et al., 2016; Wang et al., 2022a). Mutations in the members of complex I would change the metabolic capacity which may further affect adaptability of the organism (Zhao et al., 2022).

### 4.2 The different thermal acclimation in the three *Coptis* species and the physiological indices of mitochondrial complex, antioxidants and related substances

*C. chinensis*, *C. deltoidea* and *C. omeiensis* exhibit significant differences in the altitudinal distribution ranges and thermal acclimation. Currently, *C. chinensis* is the most widely cultivated and is the dominant species on the market due to its environmental adaptability and ability to grow at low altitudes. In contrast, *C. deltoidea* suitable for growing alpine and humid climatic conditions, is highly susceptible to heat stress (**Supplementary Figure 7**) and cannot be grown in areas with higher temperatures (Li et al., 2020). To understand the mechanism of the differences in heat acclimation of the three *Coptis* plants, mitochondrial genes, mitochondrial complex and antioxidase activities, and the physiological indices of related substances were compared under heat stress. The results showed that high temperature stress adversely affected the mitochondrial complex I and V, antioxidant enzyme system, ROS accumulation and ATP production of three *Coptis* species. It has been shown that abiotic stress can impair the complex I activity, while mitochondrial ROS produced during stress could occur by inhibition of the ETC (Møller, 2001; Jacoby et al., 2011). However, inhibition of complex I leads to a slowing of electron flow through the ETC and a decrease in respiration, thus inhibiting the synthesis of ATP (Rustin and Lance, 1986; Belhadj Slimen et al., 2014).

Compared to the control treatment (20°C), *C. chinensis* adapted better to high temperature through the activation of antioxidant enzymes, increase of T-AOC and maintenance of low ROS accumulation, which were suggested as the factors that maintained normal growth at lower altitudes. Nevertheless, the CAT activity and T-AOC reduced, ROS accumulation increased in the warming sensitive *C. deltoidea* under heat stress, which may indicate increased ROS production as well as diminished scavenging capacity of *C. deltoidea*. However, when ROS formation exceeds normal levels, it may cause damage to membranes and plant cell functions (Rhoads et al., 2006). The distribution altitude of *C. omeiensis* is lower than that of *C. deltoidea* and its range is narrower than that of *C. chinensis*. High temperature stress elevated the antioxidant capacity and ROS accumulation of *C. omeiensis*, but ROS accumulation was lower than that of *C. deltoidea*. Previous studies have shown that the thermotolerant cultivar greater activate antioxidant enzyme system compared to the sensitive cultivar. Increased activity of these antioxidant enzymes suppressed the levels of superoxide anion and H_2_O_2_ levels, which were otherwise high in sensitive cultivars (Xu et al., 2015; Tiwari et al., 2022). However, plants have evolved complex adaptive mechanisms to cope with environmental stress, which may involve several metabolic adjustments, gene expression and morpho-physiological alterations (Yadav et al., 2016; Tiwari and Yadav, 2019). Therefore, further studies are necessary to explore genes and molecular regulation mechanism involved in different heat acclimation in *Coptis* species to further comprehensive understanding the environmental adaptation mechanisms, which is of great significance for the breeding of heat-resistant varieties under the background of global warming and extreme heat intensification (Xu et al., 2020; Wang et al., 2022b).

## 5 Conclusions

In this study, we first assembled and characterized the complete mitochondrial genomes of *C. chinensis*, *C. deltoidea* and *C. omeiensis*, the important medicinal members of the Ranunculaceae family. The mitogenomes are 1,425,403 bp, 1,520,338 bp and 1,152,812 bp, and harbors 68-86 predicted functional genes including 39-51 PCGs, 26-35 tRNAs and 2-5 rRNAs. The codon usage, repeat sequences, RNA editing edits, gene migration from chloroplast, repeat sequences and genome rearrangement in the *Coptis* mitochondrial genomes were extensively analyzed, contributing to our understanding of conservation and variation of *Coptis* mitogenomes during the evolution. *C. chinensis*, *C. deltoidea* and *C. omeiensis* exhibit significant differences in the altitudinal distribution ranges and thermal acclimation. High temperature stress adversely affected the mitochondrial complex I and V, antioxidant enzyme system, ROS accumulation and ATP production of three *Coptis* species. *C. chinensis* with strong environmental adaptability could effectively enhance antioxidant capacity, scavenge ROS, and maintain energy supply. This study provides comprehensive information on the *Coptis* mitogenomes and is of great importance to elucidate the mitochondrial functions, understand the different thermal acclimation mechanisms of *Coptis* plants, and breed heat-tolerant varieties.

## Data availability statement

The datasets presented in this study can be found in online repositories. The names of the repository/repositories and accession number(s) can be found below: https://www.ncbi.nlm.nih.gov/genbank/, OP466716 https://www.ncbi.nlm.nih.gov/nuccore/OP466716, OP466717 https://www.ncbi.nlm.nih.gov/nuccore/OP466718, OP466718 https://www.ncbi.nlm.nih.gov/genbank/, OP466716 https://www.ncbi.nlm.nih.gov/nuccore/OP466716, OP466717 https://www.ncbi.nlm.nih.gov/nuccore/OP466717, OP466721 https://www.ncbi.nlm.nih.gov/nuccore/OP466718, OP466722 https://www.ncbi.nlm.nih.gov/nuccore/OP466722, OP466723 https://www.ncbi.nlm.nih.gov/nuccore/OP466723, OP466724 https://www.ncbi.nlm.nih.gov/nuccore/OP466724, OP466725 https://www.ncbi.nlm.nih.gov/nuccore/OP466725.

## Author contributions

FZ, ZY, YM designed the project and the strategy, YL, WK, and XC contributed to plant sample collection and processing. FZ, YL, and WK work on genome assembly, annotation and comparative analyses. FZ, YL, TZ and BX wrote and revised the manuscript. ZY and YM helped with a critical discussion on the work. All authors contributed to the article and approved the submitted version.
