# Supplementary material for: Comprehensive analysis of the complete mitochondrial genomes of three Coptis species (C. chinensis, C. deltoidea and C. omeiensis): the important medicinal plants in China
Source: Front Plant Sci. 2023 May 29;14:1166420. doi: 10.3389/fpls.2023.1166420 (PMC10258346; doi:10.3389/fpls.2023.1166420)
Supplement: Supplementary file 1 [file DataSheet_1.docx]

Supplementary Material

Comprehensive analysis of the complete mitochondrial genomes of three *Coptis* species (*C. chinensis*, *C. deltoidea* and *C. omeiensis*): the important medicinal plants in China

**Furong Zhong ^1,2,#^, Wenjia Ke^1,2,#^, Yirou Li^1,2^, Xiaoyan Chen ^1,2^, Tao Zhou ^1,2^, Binjie Xu ^3^, Luming Qi ^4,5^, Zhuyun Yan ^1,2,*^, Yuntong Ma ^1,2,*^**

*** Correspondence:**

Zhuyun Yan ([cdtcmyan@126.com](mailto:cdtcmyan@126.com));

Yuntong Ma ([Mayuntong@cdutcm.edu.cn](mailto:Mayuntong@cdutcm.edu.cn))

# Supplementary Figures and Tables

## Supplementary Figures


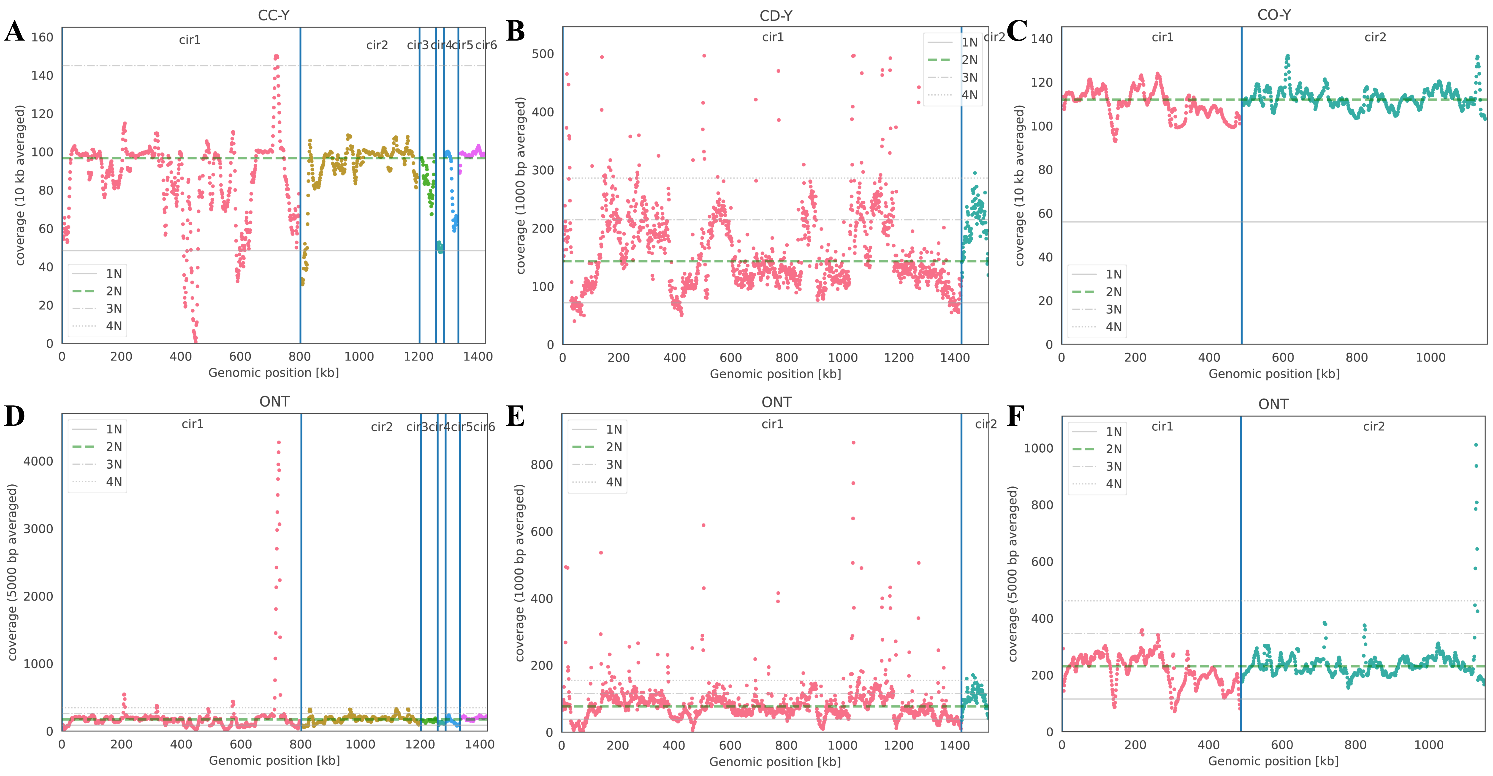


**Supplementary Figure S1** The coverage of the three newly assembled Coptis mitogenomes. (A-C) The coverage plots of Illumina short sequences of *C. chinensis*, *C. deltoidea* and *C. omeiensis*. (D-E) The coverage plots of ONT sequences of *C. chinensis*, *C. deltoidea* and *C. omeiensis*.

Note: To check the quality of the three assemblies, the illumina short sequences and ONT sequences were compared to the mitochondrial genomes using BWA software and the coverage was counted using samtools depth and plotted with mitochondrial length as the horizontal coordinate and coverage depth as the vertical coordinate. The results showed that the assemblies of *C. deltoidea* and *C. omeiensis* had high coverage. Perhaps due to the influence of material status, one location reads of *C. chinensis* assemblies were not detected base on Illumina sequencing, and the depth map showed a breakpoint at the location, but this problem was solved in the ONT sequencing. The coverage of the ONT sequencing data of *C. chinensis* mitogenome was more than 100X, and the sequencing quality was reliable with no breakpoint in coverage.


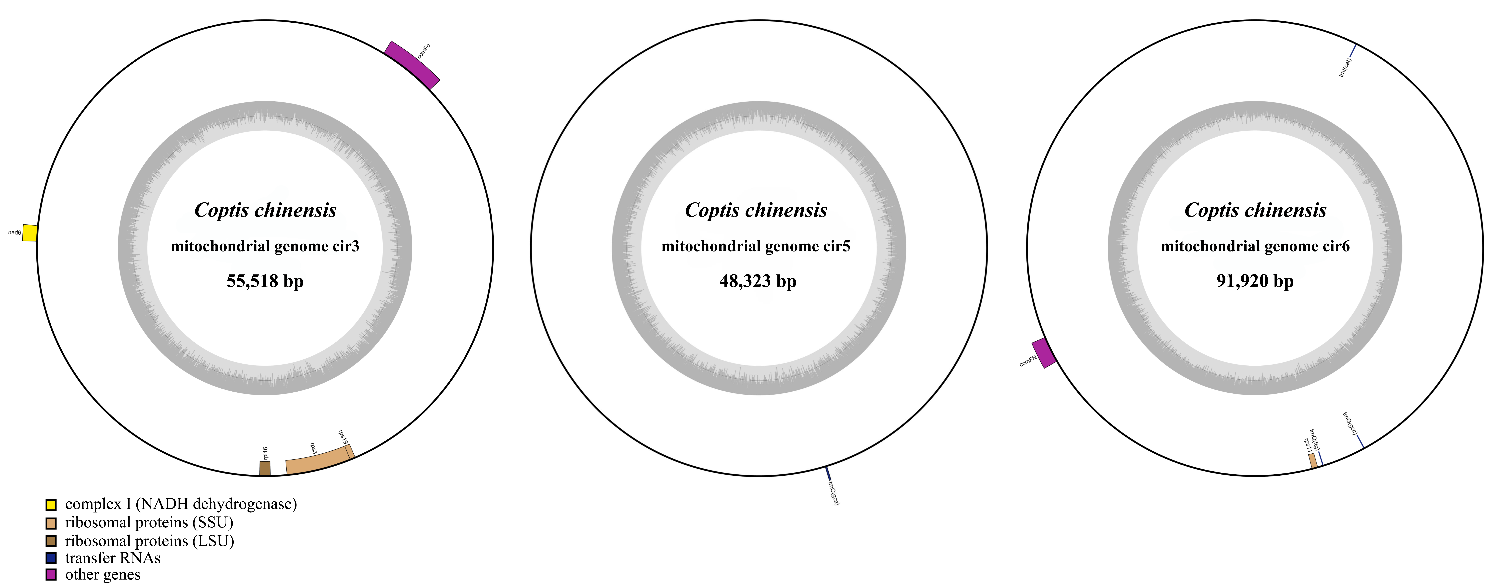


**Supplementary Figure S2** Other three circular molecules of *C. chinensis* mitogenome.


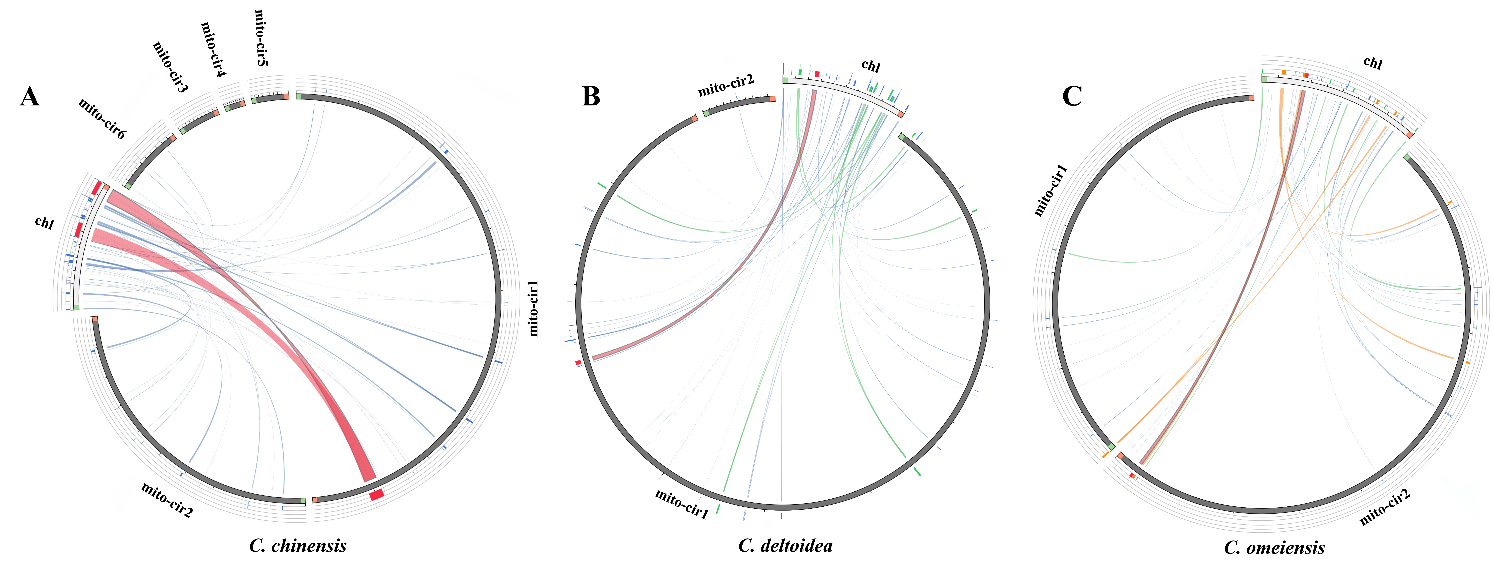


**Supplementary Figure S3** Gene transfers between chloroplast genomes and mitochondrial genomes of C. chinensis (A), *C. deltoidea* (B) and *C. omeiensis* (C). The color of the connecting line is selected according to ratio = score/max, and the score is differently colored in the corresponding range blue ≤ 0.25, green ≤ 0.50, orange ≤ 0.75, red > 0.75. chl for chloroplast and mito for mitochondria.


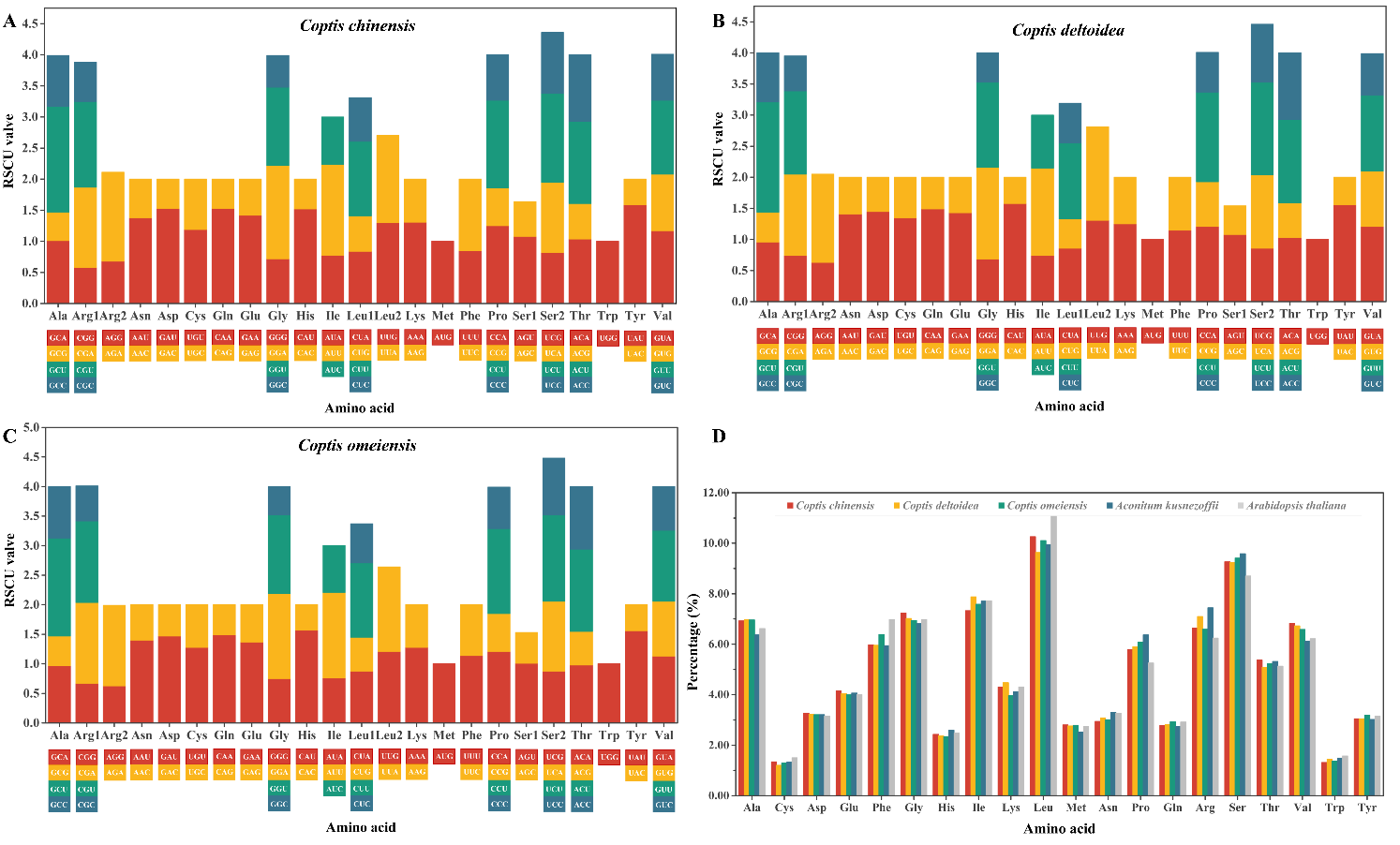


**Supplementary Figure S4** Relative synonymous codon usage (RSCU) (A-C) and Codon usage pattern (D) in the three *Coptis* mitochondrial genomes.


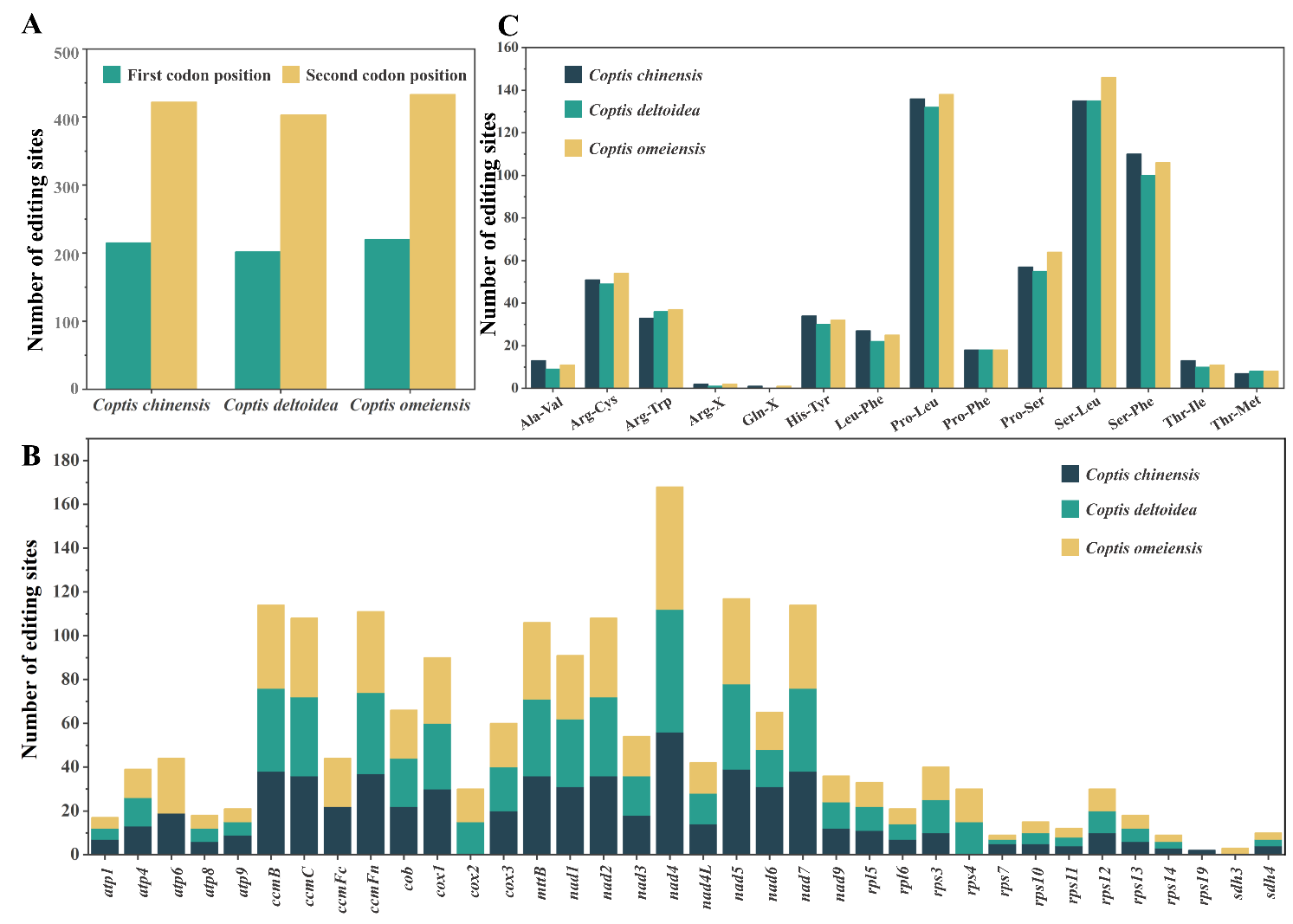


**Supplementary Figure S5** RNA editing sites in mitogenome protein coding genes of three *Coptis* species. (A) Number of RNA editing sites at the first and second codon positions. (B) Number of RNA editing sites of protein coding genes. (C) Number of RNA editing sites leading to different amino acid transformations.


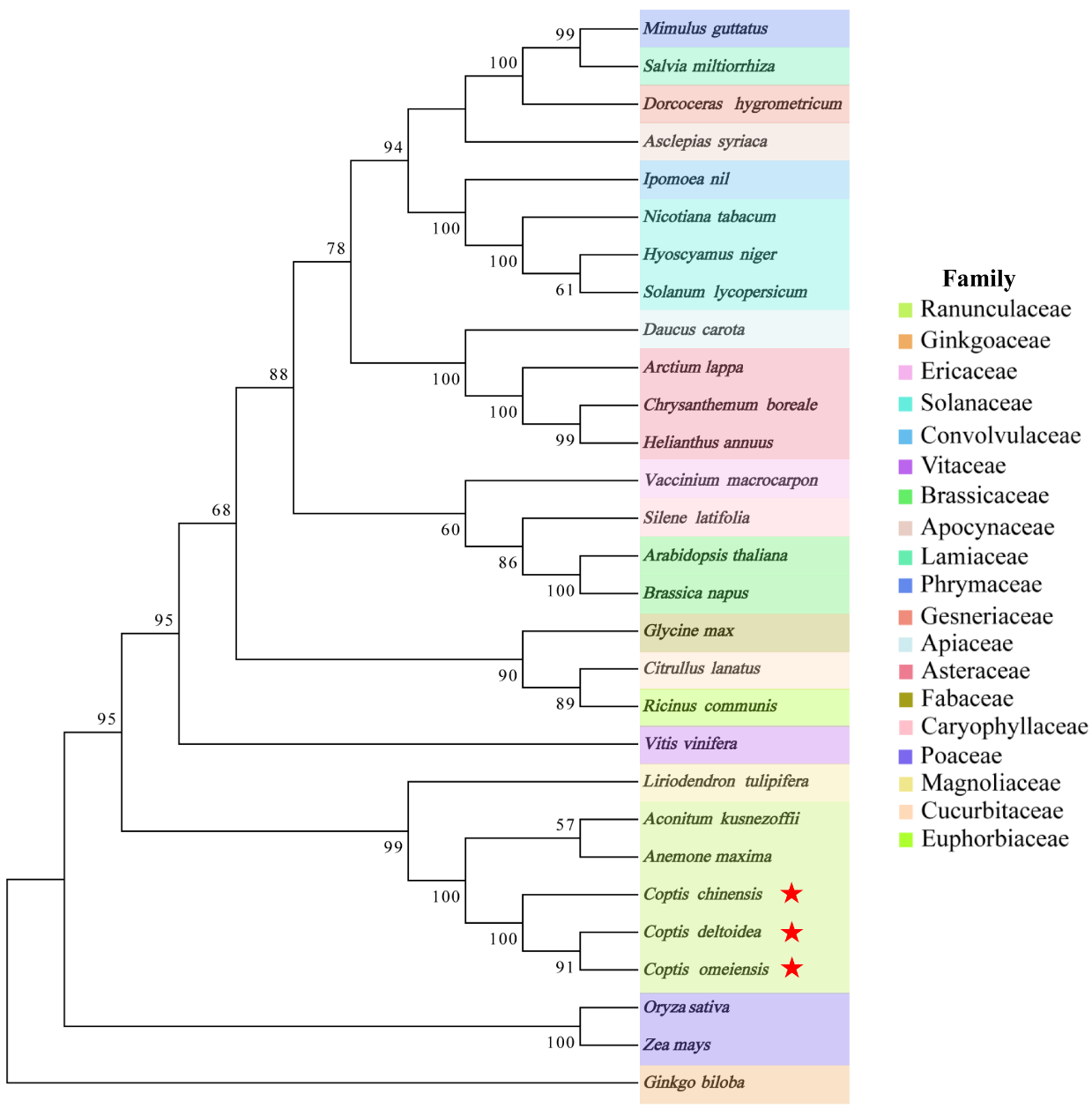


**Supplementary Figure S6** The phylogenetic relationships of three *Coptis* species with other 26 plant species. The Maximum Likelihood tree was constructed based on the sequences of 23 conserved protein-coding genes. Colors indicate the families that the specific species belongs.


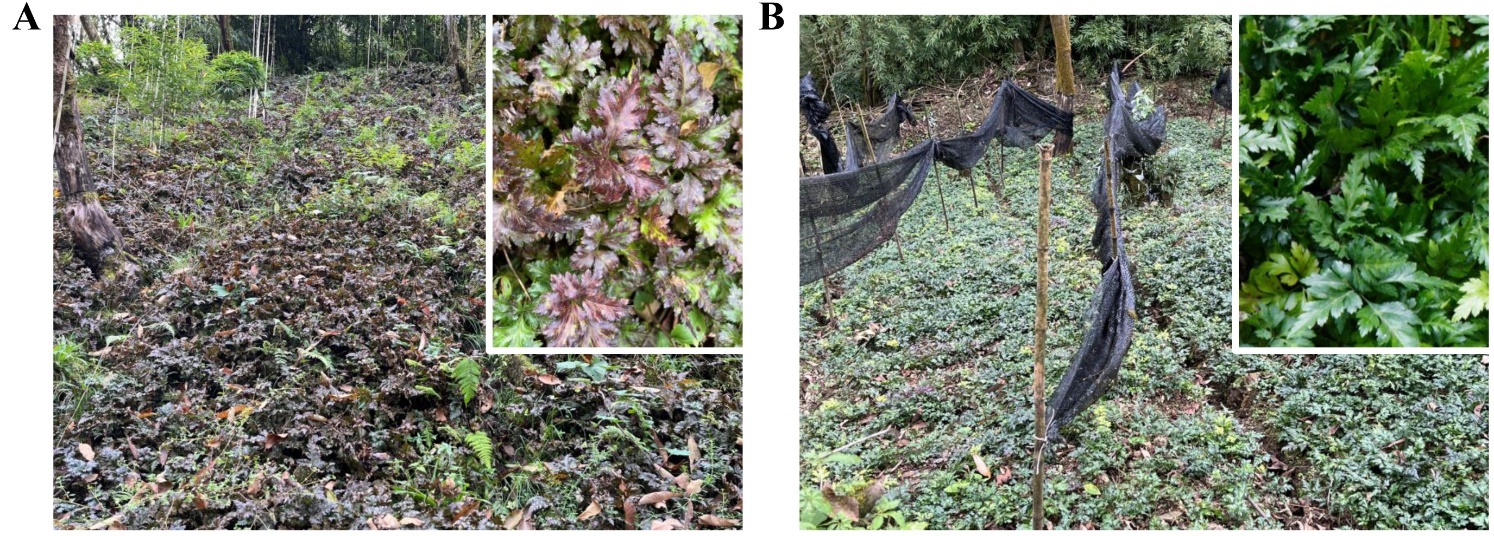


**Supplementary Figure S7** Plants of *C. deltoidea* (A) and *C. chinensis* (B) under the extreme high temperature weather in summer.

## Supplementary Tables

**Supplementary Table S1.** Details regarding the mitochondrial genomes used for the phylogenetic analysis.

| No. | Taxon | Family | Order | GenBank Accession number |
| --- | --- | --- | --- | --- |
| 1 | *Mimulus guttatus* | Phrymaceae | Lamiales | NC_018041.1 |
| 2 | *Salvia miltiorrhiza* | Lamiaceae | Lamiales | NC_023209.1 |
| 3 | *Dorcoceras hygrometricum* | Gesneriaceae | Lamiales | NC_016741.1 |
| 4 | *Asclepias syriaca* | Apocynaceae | Gentianales | NC_022796.1 |
| 5 | *Ipomoea nil* | Convolvulaceae | Solanales | NC_031158.1 |
| 6 | *Nicotiana tabacum* | Solanaceae | Solanales | NC_006581.1 |
| 7 | *Hyoscyamus niger* | Solanaceae | Solanales | NC_026515.1 |
| 8 | *Solanum lycopersicum* | Solanaceae | Solanales | NC_035963.1 |
| 9 | *Daucus carota* | Apiaceae | Apiales | NC_017855.1 |
| 10 | *Arctium lappa* | Asteraceae | Asterales | MZ475019.1 |
| 11 | *Chrysanthemum boreale* | Asteraceae | Asterales | NC_039757.1 |
| 12 | *Helianthus annuus* | Asteraceae | Asterales | NC_023337.1 |
| 13 | *Vaccinium macrocarpon* | Ericaceae | Solanales | NC_023338.1 |
| 14 | *Silene latifolia* | Caryophyllaceae | Caryophyllales | NC_014487.1 |
| 15 | *Arabidopsis thaliana* | Brassicaceae | Brassicales | NC_037304.1 |
| 16 | *Brassica napus* | Brassicaceae | Brassicales | NC_008285.1 |
| 17 | *Glycine max* | Fabaceae | Fabales | NC_020455.1 |
| 18 | *Citrullus lanatus* | Cucurbitaceae | Cucurbitales | NC_014043.1 |
| 19 | *Ricinus communis* | Euphorbiaceae | Euphorbiales | NC_015141.1 |
| 20 | *Vitis vinifera* | Vitaceae | Vitales | NC_012119.1 |
| 21 | *Liriodendron tulipifera* | Magnoliaceae | Magnoliales | NC_021152.1 |
| 22 | *Magnolia officinalis* | Magnoliaceae | Magnoliales | NC_064401.1 |
| 23 | *Magnolia biondii* | Magnoliaceae | Magnoliales | NC_049134.1 |
| 24 | *Pulsatilla chinensis* | Ranunculaceae | Ranunculales | NC_068017.1 |
| 25 | *Pulsatilla dahurica* | Ranunculaceae | Ranunculales | NC_071219.1 |
| 26 | *Aconitum kusnezoffii* | Ranunculaceae | Ranunculales | NC_053920.1 |
| 27 | *Anemone maxima* | Ranunculaceae | Ranunculales | NC_053368.1 |
| 28 | *Oryza sativa* | Poaceae | Poales | NC_007886.1 |
| 29 | *Zea mays* | Poaceae | Poales | NC_007982.1 |
| 30 | *Ginkgo biloba* | Ginkgoaceae | Ginkgoales | NC_027976.1 |

**Supplementary Table S2.** Real-time quantitative PCR primers used in this study.

| Gene name | Primer name | Primer sequence | species |
| --- | --- | --- | --- |
| *nad1* | nad1-F | ATATATGGGTCCGTGCAGCATTTCC | *C. chinensis, C. deltoidea C. omeiensis* |
|  | nad1-R | AAGGGAGCCATTGAAAGGTGACTG |  |
| *nad2* | nad2-F | CTTTGGGTTGTGGGGCTTACTTCC | *C. chinensis*  *C. omeiensis* |
|  | nad2-R | ACGACTTGTCACGATCCATTGGTTC |  |
|  | Cdnad2-F | AGCGGTTTCCCCAGAGATCTTTATC | *C. deltoidea* |
|  | Cdnad2-R | ATCCAAGCCAACCCACATTACTGAC |  |
| *nad5* | nad5-F | TTGCTTTCTGGTTGGGAAGTGTCTC | *C. chinensis, C. deltoidea C. omeiensis* |
|  | nad5-R | TGTCTCGCCCGAATGAATTAGTTGG |  |
| *nad6* | nad6-F | TCCTGCTTTGGTCTCTGGTTTGATG | *C. chinensis, C. deltoidea C. omeiensis* |
|  | nad6-R | GCGAAAGACTGGGATGGGAAACG |  |
|  | Cdatp1-R | TCGAGCTACCTGCACCTGTCTG |  |

**Supplementary Table S3.** Nanopore sequencing results.

| Sample | Raw sequence Number | Clean sequence Number | Raw base (G) | Clean Base (G) | Q20  (%) | Q30  (%) | GC Content  (%) |
| --- | --- | --- | --- | --- | --- | --- | --- |
| *C. chinensis* | 2132738 | 1967840 | 11.3 | 11.22 | 96.84 | 91.72 | 38.63 |
| *C. deltoidea* | 943394 | 871820 | 10.77 | 10.69 | 96.91 | 91.77 | 38.91 |
| *C. omeiensis* | 1799160 | 1675626 | 11.26 | 11.19 | 96.81 | 91.54 | 38.30 |

**Supplementary Table S4.** Illumina PE sequencing results.

| Sample | Raw reads | Clean reads | Raw base (G) | Clean Base (G) | Q20  (%) | Q30  (%) | GC Content  (%) |
| --- | --- | --- | --- | --- | --- | --- | --- |
| *C. chinensis* | 37679149 | 37400775 | 11.3 | 11.22 | 96.84 | 91.72 | 38.63 |
| *C. deltoidea* | 35885113 | 35632911 | 10.77 | 10.69 | 96.91 | 91.77 | 38.91 |
| *C. omeiensis* | 37523528 | 37307791 | 11.26 | 11.19 | 96.81 | 91.54 | 38.30 |

**Supplementary Table S5.** Genomic features of *C. chinensis*, *C. deltoidea* and *C. omeiensis* mitogenomes.

| Sample | | Genome Size (bp) | A % | C % | G % | T % | GC % | Accession no. |
| --- | --- | --- | --- | --- | --- | --- | --- | --- |
| *C. chinensis* | cir1 | 802323 | 27.2 | 22.9 | 22.8 | 27.1 | 45.7 | OP466716 |
|  | cir2 | 400678 | 27.1 | 22.8 | 22.8 | 27.3 | 45.6 | OP466717 |
|  | cir3 | 55518 | 26.4 | 23.0 | 23.0 | 27.6 | 46.0 | OP466718 |
|  | cir4 | 26641 | 29.2 | 21.5 | 21.5 | 27.8 | 43.0 | OP466719 |
|  | cir5 | 48323 | 27.4 | 22.6 | 23.3 | 26.7 | 45.9 | OP466720 |
|  | cir6 | 91920 | 26.6 | 22.8 | 23.2 | 27.4 | 46.0 | OP466721 |
| *C.* *deltoidea* | cir1 | 1422914 | 27.0 | 22.9 | 22.9 | 27.2 | 45.8 | OP466722 |
|  | cir2 | 97424 | 27.1 | 23.0 | 22.3 | 27.5 | 45.4 | OP466723 |
| *C. omeiensis* | cir1 | 488072 | 26.9 | 23.1 | 22.7 | 27.3 | 45.8 | OP466724 |
|  | cir2 | 664740 | 27.1 | 23.1 | 22.7 | 27.1 | 45.8 | OP466725 |

**Supplementary Table S6.** Prediction of RNA editing sites.

| Type | RNA-editing | *Coptis chinensis* | | *Coptis deltoidea* | | *Coptis omeiensis* | |
| --- | --- | --- | --- | --- | --- | --- | --- |
|  |  | Number | Percentage | Number | Percentage | Number | Percentage |
| hydrophobic | GCA (A) => GTA (V) | 1 | 30.46% | 1 | 29.92% | 1 | 29.71% |
|  | GCG (A) => GTG (V) | 7 |  | 5 |  | 6 |  |
|  | GCT (A) => GTT (V) | 5 |  | 3 |  | 4 |  |
|  | CTC (L) => TTC (F) | 10 |  | 8 |  | 9 |  |
|  | CTT (L) => TTT (F) | 17 |  | 14 |  | 16 |  |
|  | CCC (P) => TTC (F) | 6 |  | 6 |  | 6 |  |
|  | CCT (P) => TTT (F) | 12 |  | 12 |  | 12 |  |
|  | CCA (P) => CTA (L) | 54 |  | 54 |  | 55 |  |
|  | CCC (P) => CTC (L) | 12 |  | 11 |  | 13 |  |
|  | CCG (P) => CTG (L) | 39 |  | 38 |  | 41 |  |
|  | CCT (P) => CTT (L) | 31 |  | 29 |  | 31 |  |
| hydrophilic | CAC (H) => TAC (Y) | 9 | 13.34% | 9 | 13.06% | 9 | 13.17% |
|  | CAT (H) => TAT (Y) | 25 |  | 21 |  | 23 |  |
|  | CGC (R) => TGC (C) | 13 |  | 13 |  | 13 |  |
|  | CGT (R) => TGT (C) | 38 |  | 36 |  | 41 |  |
| hydrophobic-hydrophilic | CCA (P) => TCA (S) | 13 | 8.95% | 12 | 9.09% | 13 | 9.49% |
|  | CCC (P) => TCC (S) | 13 |  | 13 |  | 15 |  |
|  | CCG (P) => TCG (S) | 7 |  | 8 |  | 9 |  |
|  | CCT (P) => TCT (S) | 24 |  | 22 |  | 25 |  |
| hydrophilic-hydrophobic | CGG (R) => TGG (W) | 33 | 46.78% | 36 | 47.77% | 37 | 47.17% |
|  | TCC (S) => TTC (F) | 44 |  | 40 |  | 44 |  |
|  | TCT (S) => TTT (F) | 66 |  | 60 |  | 62 |  |
|  | TCA (S) => TTA (L) | 81 |  | 82 |  | 90 |  |
|  | TCG (S) => TTG (L) | 54 |  | 53 |  | 56 |  |
|  | ACA (T) => ATA (I) | 5 |  | 5 |  | 6 |  |
|  | ACC (T) => ATC (I) | 1 |  | 1 |  | 1 |  |
|  | ACT (T) => ATT (I) | 7 |  | 4 |  | 4 |  |
|  | ACG (T) => ATG (M) | 7 |  | 8 |  | 8 |  |
| hydrophilic-stop | CGA (R) => TGA (X) | 2 | 0.47% | 1 | 0.16% | 2 | 0.46% |
|  | CAG (Q) => TAG (X) | 1 |  | 0 |  | 1 |  |

**Supplementary Table S7.** Synteny blocks of the three *Coptis* mitogenomes.

| **Species compared** | | **Genome size** | | **Synteny blocks** | | | | |
| --- | --- | --- | --- | --- | --- | --- | --- | --- |
| Species 1 | Species 2 | Species 1 | Species 2 | S1 to S2 | S2 to S1 | % genome S1 | % genome S2 | Block numbers |
| *C. chinensis* | *C. deltoidea* | 1,425,403 bp | 1,520,338 bp | 418,907 bp | 418,901 bp | 29.39 | 27.55 | 129 |
| *C. chinensis* | *C. omeiensis* | 1,425,403 bp | 1,152,812 bp | 708,034 bp | 708,207 bp | 49.67 | 61.42 | 246 |
| *C. omeiensis* | *C. deltoidea* | 1,152,812 bp | 1,520,338 bp | 505,284 bp | 505,249 bp | 46.33 | 35.12 | 34 |
